# MOFI-FL, a novel score for detecting hepatic steatosis and predicting cardiometabolic mortality

**DOI:** 10.1038/s41598-025-15487-7

**Published:** 2025-08-19

**Authors:** Juan Reyes-Barrera, Rosalinda Posadas-Sánchez, Gilberto Vargas-Alarcón, Guillermo C. Cardoso-Saldaña, Paloma Almeda-Valdes, Omar Yaxmehen Bello Chavolla, Luis Ortiz-Hernandez, Neftali Eduardo Antonio-Villa

**Affiliations:** 1 Doctorado en Ciencias Biológicas y de la Salud, Universidad Autónoma Metropolitana, Ciudad de México, México; 2 Department of Endocrinology, Instituto Nacional de Cardiología Ignacio Chávez, Juan Badiano Nº 1, Colonia Sección XVI, México City, C. P. 14030 México; 3 Department of Molecular Biology, Instituto Nacional de Cardiología Ignacio Chávez, México City, México; 4 Research Direction, Instituto Nacional de Cardiología Ignacio Chávez, México City, México; 5 Endocrinology and Metabolism Department, Instituto Nacional de Ciencias Médicas y Nutrición Salvador Zubirán, Mexico City, México; 6 Metabolic Research Unit, Instituto Nacional de Ciencias Médicas y Nutrición Salvador Zubirán, Mexico City, México; 7 Division of Research, Instituto Nacional de Geriatría, México City, México; 8 Department of Global Health and Population, Harvard T.H. Chan School of Public Health, Boston, MA USA; 9 Departamento de Atención a la Salud, Universidad Autónoma Metropolitana Unidad Xochimilco, Ciudad de México, México

**Keywords:** Liver disease, Hepatic steatosis, Fatty liver, Clinical surrogate, Biomarkers, Cardiology, Risk factors, Endocrinology, Endocrine system and metabolic diseases

## Abstract

Hepatic steatosis (HS) is a common condition linked to increased cardiometabolic risk; however, biopsy and imaging-based methods limit the widespread diagnosis, especially in low-resource settings. Here, we develop the MOFI-FL index, a simplified tool for HS detection, validate its performance against vibration-controlled transient elastography (VCTE), and evaluate its association with all-cause and cause-specific mortality. Using data from the Genetics of Atherosclerotic Disease study as our discovery cohort, we developed MOFI-FL, incorporating ALT, glucose, uric acid, and BMI, with computed tomography (CT) as the gold standard of HS. We used the continuous NHANES (2017–2018 cycles,* n* = 4,405) to validate against VCTE and compare our index with four previously validated HS indices (FLI, HSI, NAFLD-LFS, and AST/ALT). Finally, we assessed all-cause and cause-specific mortality prediction using the NHANES-III cohort (*n* = 12,684) using Cox proportional hazards models adjusted for relevant confounders. The MOFI-FL index demonstrated good diagnostic performance in the internal validation cohort against CT (AUROC: 0.78 [95% CI: 0.72–0.83]; accuracy: 75% [70–79]) and against VCTE (NHANES 17–18: AUROC 0.77 [0.68–0.76]; accuracy: 70% [68–71]). It outperformed existing HS indices in the external cohort. Furthermore, a 1% increase in MOFI-FL was positively associated with all-cause mortality (HR = 1.005 [1.004–1.007]), as well as cardiovascular (HR = 1.008 [1.004–1.007]), diabetes-related (HR = 1.034 [1.028–1.040]), and nephrological deaths (HR = 1.012 [1.000–1.024]). MOFI-FL is a novel and simple tool for HS detection. It offers comparable performance to established indices and predictive capacity for cardiometabolic mortality, making it accessible for clinical and epidemiological applications.

**Supplementary Information:**

The online version contains supplementary material available at 10.1038/s41598-025-15487-7.

## Introduction

Hepatic steatosis (HS) is the most common chronic liver disease, with a worldwide estimated prevalence of 30%^1^. HS disproportionately affects individuals in low- and middle-income countries (LMICs) compared to more developed regions. Specifically, in Latin America the prevalence of HS is estimated at 44%^2,3^, yielding it as a condition of high epidemiological burden. HS is characterized by ectopic adipose tissue deposition in the liver and is often asymptomatic, making early diagnosis challenging^4,5^. Moreover, HS is closely linked to obesity, type 2 diabetes (T2D), and metabolic syndrome (MS), all of which contribute to an increased risk of cardiovascular disease (CVD). Thus, prompt HS diagnosis is crucial to mitigate its cardiometabolic risk^6^.

Diverse methods can be used to detect HS; nevertheless, it’s an area of constant debate^7–9^. Despite its invasive nature, potential for sampling error, and low inter-rater reliability, liver biopsy remains the gold standard for diagnosing HS and staging liver fibrosis. However, these limitations restrict its widespread application in resource-limited settings and within epidemiological studies^10^. Therefore, simple, cost-effective, and validated non-invasive screening tools are essential for identifying individuals with high risk of HS in clinical practice and epidemiological studies. Various non-invasive tools, including imaging-based methods (e.g., tomography-computed [CT], magnetic resonance imaging [MRI], and ultrasonography [USG]) and clinical surrogates indices (e.g., fatty liver index [FLI], hepatic steatosis index [HS], NAFLD liver fat score [NAFLD-LFS], and AST/ALT ratio), have been developed to screen for fatty liver, and minimize the need for biopsy procedures^8,11^. Imaging-based methods have proven to be good diagnostic tools for fatty liver. However, these are operator-dependent and are generally impractical for repeated assessments or large-scale population studies^12^.

Clinical surrogates have emerged as simple and cost-effective tools to identify widespread cardiometabolic conditions including HS, particularly in LMICs settings^13^. However, some clinical surrogates include biochemical measurements that are not routinely determined (e.g., insulin, apolipoprotein-a, or haptoglobin) or previous diagnostics constructs (e.g., T2D, hypertension and MS) that require a more profound clinical evaluation^14^. Despite the widespread use of non-invasive indices such as the FLI, HSI, NAFLD-LFS, and AST/ALT ratio, their diagnostic performance remains inconsistent across various populations and diagnostic methods. Additionally, there is a lack of external validation in many ethnic groups, which may limit the generalizability and clinical applicability of these indexes in various settings^15,16^. In this context, the FLI exhibits variable diagnostic performance, depending on the risk profile of the population, with lower accuracy observed in individuals with metabolic syndrome or high cardiometabolic risk^17,18^. Furthermore, the HSI performs poorly in populations with metabolic syndrome and includes a prior diabetes diagnosis as part of its criteria, which limits its effectiveness for early detection of hepatic steatosis in non-diseased individuals^18–20^. The NAFLD-LFS requires insulin measurements, which are not routinely available in many clinical settings; moreover, its specificity and overall performance differ significantly across various risk profiles. Finally, the AST/ALT ratio is more indicative of advanced liver disease or fibrosis, and is not a reliable tool for detecting early hepatic steatosis^17,19,20^.

Thus, there is an opportunity to develop clinical surrogates that could assist clinicians in detecting HS. A novel index could be integrated within primary care and public health personnel to offer accessible and cost-effective strategies for mitigating HS, particularly in LMIC settings.

In this study, we aimed to (1) develop a simplified index to detect HS, (2) validate this index within an external cohort and compare its performance with existing indices, and (3) determine whether our novel index could predict all-cause and cause-specific mortality in a nationally representative health survey.

## Methods

### Study design and data sources

#### Study overview

**Fig. 1:**
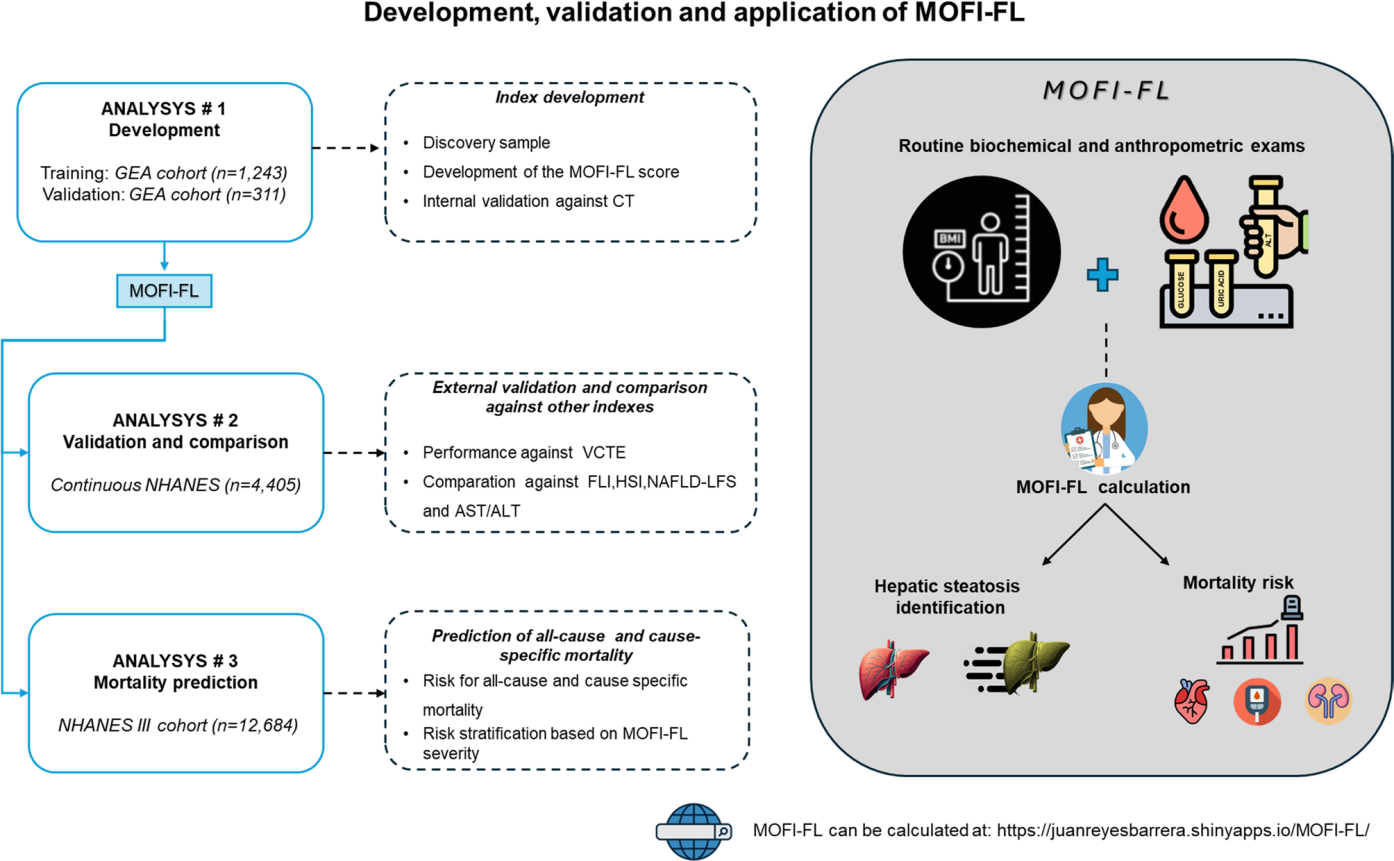
Flowchart diagram of development, validation, and application of MOFI-FL, along with key findings from our study. On the right, a conceptual framework summarizes how MOFI-FL is formed and its capability to identify HS and predict cardiometabolic mortality. To estimate MOFI-FL, please visit: https://juanreyesbarrera.shinyapps.io/MOFI-FL/. *Abbreviations*: MOFI-FL: model for identification of fatty liver, HS: hepatic steatosis, CT: computed tomography, VCTE: vibration-controlled transient elastography, FLI: fatty liver index, HSI: hepatic steatosis index, NAFLD-LFS: non-alcoholic fatty liver disease liver fat score, AST/ALT: aspartate aminotransferase/alanine aminotransferase ratio, GEA: Genetics of Atherosclerotic Disease, *Continuous NHANES*: National Health and Nutrition Examination Survey 2017–2018 cycle, NHANES III: National Health and Nutrition Examination Survey 1988–1994 cycle,

A diagram of our study design is presented in Figure 1. Briefly, we used three datasets as follows: The Genetics of Atherosclerotic Disease (GEA) study served as the discovery sample for index development^21^. We then applied our index to the National Health and Nutrition Examination Survey 2017–2018 cycle (*continuous NHANES* )^22^. Finally, we used the NHANES-III survey to predict all-cause and cause-specific mortality. Expanded methods and ethics approval (where applicable) for each cohort are presented in **Supplementary Table 1**. This study adheres to Standards for Reporting of Diagnostic Accuracy Studies (STARD) guidelines^23^ (**Supplementary Table 2**). This study was approved by the Research and Ethics Committee of the National Institute of Cardiology Ignacio Chavez (Protocol Number: 09–646).

## Discovery sample

The discovery sample was drawn from the control group of the GEA study. Briefly, the GEA study is a community-based cohort of adults designed to investigate the genomic basis of premature coronary artery disease (CAD) and its relationship with traditional and emerging risk factors in the Mexican population^21^. The GEA control group recruited individuals aged 30–75 years with no clinical or family history of premature CAD. For this analysis, we excluded participants who had significant alcohol consumption and incomplete CT measurements. The gold standard for identifying subjects with HS and developing our index was the use of non-contrast CT^8^. HS was defined by a liver-to-spleen attenuation ratio < 1.0, using a 64-slice scanner (Somatom Cardiac Sensation; Medical Solutions, Forchheim Germany)^24^. Expanded methods of anthropometric, biochemical, and image techniques of the GEA study are presented in **Supplementary Table 3**.

## External validation cohort

- *Continuous NHANES*: Participants were excluded if they lacked vibration-controlled transient elastography (VCTE) or controlled attenuation parameter (CAP) measurements, had hepatitis B or C, had significant alcohol consumption, or used steatogenic drugs (e.g. such as amiodarone, methotrexate, and tamoxifen). In the *Continuous NHANES*, HS was evaluated using a FibroScan 502 Touch device to perform VCTE. HS was defined as CAP ≥ 285 dB/m^25^.

## Prediction mortality cohort

- *NHANES-III*: We analyzed data from NHANES-III (1988–1994)^26^, with linked information to the National Death Index, which provides a follow-up from the initial examination until death or censoring on December 31, 2022^27^. For the present analysis, we included participants aged between 20 and 85 years with complete clinical and mortality data.

### Development and validation of the index

#### Mathematical modeling

To develop our index, we first identified key variables from the GEA study that served as key HS predictors. Based on previous research^28–32^, we included the following variables that could be optimal predictors of HS: age, sex, alanine transaminase (ALT), aspartate aminotransferase (AST), gamma-glutamyl transferase (GGT), fasting glucose, fasting triglycerides, waist circumference (WC), uric acid, total cholesterol, body mass index (BMI), high-density lipoprotein cholesterol (HDL-C), serum adiponectin, high-sensitive C-reactive protein (hs-CRP), fasting insulin, and low-density lipoprotein cholesterol (LDL-C). The technique of each biochemical measurement is presented in **Supplementary Table 3**. Logarithmic transformations (natural logarithm, base *e*) were applied to approximate normality in variables showing non-symmetric distribution. We randomly divided the sample from the GEA study into a training set (80%) and a testing set (20%) for internal validation, this approach assigned a consistent proportion of individuals to each set. In our training set, we evaluated combinations of these predictors using binomial logistic regression models to identify the model that best predicts the probability of an individual having HS based on the Akaike Information Criterion (AIC) minimization. We selected the top 10 candidate models for further consideration based on AIC minimization. Next, we assessed the importance of each variable and manually refined the final model by prioritizing variables that were both influential and more accessible in low-resource settings (e.g., excluding those that included fasting insulin, serum adiponectin, and hs-CRP), which we termed simplified model. The resulting equation was termed MOFI-FL (*Model for Identification of Fatty Liver*). We further extracted the predicted probability of MOFI-FL and expressed it in percentage probability from 0 to 100%. Finally, we examined the correlation of MOFI-FL with serum adiponectin, fasting insulin, and visceral adipose tissue levels as key physiological metrics measured from the GEA study.

## Validation of MOFI-FL against other HS indexes

We further compared MOFI-FL with established indexes to detect HS, including the FLI, HSI, the NAFLD-LFS, and the AST/ALT ratio. These indices were calculated as follows:

- **FLI**^33^ = ((10 × triglycerides [mg/dL] + BMI [kg/m²] + GGT [U/L] + waist circumference [cm]) ÷ 2)
- **HSI**^34^= (8 × ALT/AST ratio + BMI [kg/m²] + 2 [if female])
- **NAFLD Liver Fat Score**^35^= -2.89 + 1.18 × (MS: yes = 1/no = 0) + 0.45 × (T2D: yes = 1/no = 0) + 0.15 × fasting insulin [mU/L] + 0.04 × AST [U/L] − 0.94 × AST/ALT ratio.
- **AST/ALT ratio**^36^= (AST [U/L] ÷ ALT [U/L])

### Prediction of all-cause and cause-specific mortality

Finally, we used the NHANES-III dataset to evaluate the predictive capacity of MOFI-FL to predict all-cause and cause-specific mortality. All-cause mortality included death from any cause, whereas cause-specific mortality encompassed deaths due to cardiovascular disease, cerebrovascular disease, diabetes, nephrological conditions, chronic lower respiratory tract diseases, and malignant neoplasms. Time-to-event follow-up (in person-months) was calculated from the initial interview date until the participant’s last recorded visit or the date of death, whichever occurred first.

To further assess the prognostic performance of MOFI-FL, we conducted additional comparative analyses using Cox proportional hazards regression models to estimate hazard ratios (HRs) per one standardized deviation increment. MOFI-FL was also compared against other established HS indices (FLI, HSI, NAFLD-LFS, and the AST/ALT ratio). For this comparison, the sample was restricted to participants with complete data to estimate all indices. The predictive performance of each index for all-cause mortality was evaluated using the concordance index (C-index) derived from Cox proportional hazards models. The univariate model and plus two models were constructed for this purpose: Model 1 was adjusted for age and sex, while Model 2 included additional adjustments for ethnicity and number of comorbidities. This approach allowed for a robust and clinically relevant comparison of the discriminatory ability of each index.

### Statistical analyses

Continuous variables were summarized as mean ± standard deviation or median (interquartile range), according to their distribution evaluated through the Anderson-Darling normality test. Categorical variables were expressed as absolute frequencies and percentages. The missing data in the discovery sample was imputed using a multiple imputation by chained equations (MICE)^37^. It was assumed that the data were missing completely at random to handle missing values, which accounted for less than 5% of the total dataset. We generated five imputed datasets, carrying out a maximum of five iterations and combining them according to Rubin’s rules. Further details on missing data assessment and imputation are presented in **Supplementary Figure 1**. All statistical analyses were conducted in RStudio (R version 4.2.3), and the code to reproduce these results is publicly available at: https://github.com/REYJUA12235/MOFI-FL-manuscript. A two-sided p-value < 0.05 was considered as our statistically significant threshold. In **Supplementary Table 4**, we present the R packages used for the analysis.

### Calibration, cross-validation, and correlation analyses

To identify the best predictive model for HS in the discovery sample, we selected variables of the best predictive models to create the MOFI-FL index. Then, we compared its predicted probabilities to those of the best model based on AIC minimization using Bland-Altman plots and intercorrelation analyses. Next, we perform a 10-fold cross-validation for internal validation. After cross-validation, calibration curves and a Hosmer-Lemeshow test were used to evaluate calibration alignment between predicted and observed outcomes. Additionally, decision curve analysis was performed to quantify the net clinical benefit of MOFI-FL across different threshold probabilities. Spearman’s correlation coefficients and their 95% confidence intervals were calculated to explore the relationship between the predicted probabilities of MOFI-FL and key physiological measures.

### Validation and performance metrics

To validate and compare the MOFI-FL index against other HS indices and assess the contribution of MOFI-FL individual components, we used the area under the receiver operating characteristic curve (AUROC). We estimated various performance and diagnostic metrics across cohorts, including accuracy, sensitivity, specificity, positive predictive value (PPV), negative predictive value (NPV), positive likelihood ratio (LR+), negative likelihood ratio (LR-), Nagelkerke’s R², deviance, observed/expected ratio, Brier Score, and the Bayesian Information Criterion (BIC) to evaluate the discriminative ability and model fit. Additionally, we performed decision curve analyses to assess the clinical utility and net benefit of the MOFI-FL index compared to other HS indices and their individual components.

### Prediction of all-cause and cause-specific mortality

To analyze the association of MOFI-FL with all-cause mortality and cause-specific mortality, we used Cox proportional hazards regression models adjusted by age, sex, ethnicity, and number of chronic comorbidities. We used the continuous probability of the MOFI-FL and categorized it into four probability groups: <25% (low), 26–50% (medium), 51–75% (high), and > 75% (very-high). The low-probability group served as the reference category. The proportional hazard assumption was evaluated using Schoenfeld residuals. As a secondary analysis, we estimated hazard ratios (HRs) per one standardized deviation increase for MOFI-FL and other hepatic steatosis (HS) indices. The discriminatory performance of each index was evaluated using the concordance index (C-index) derived from Cox proportional hazards models.

## Results

### Study population

**Fig. 2:**
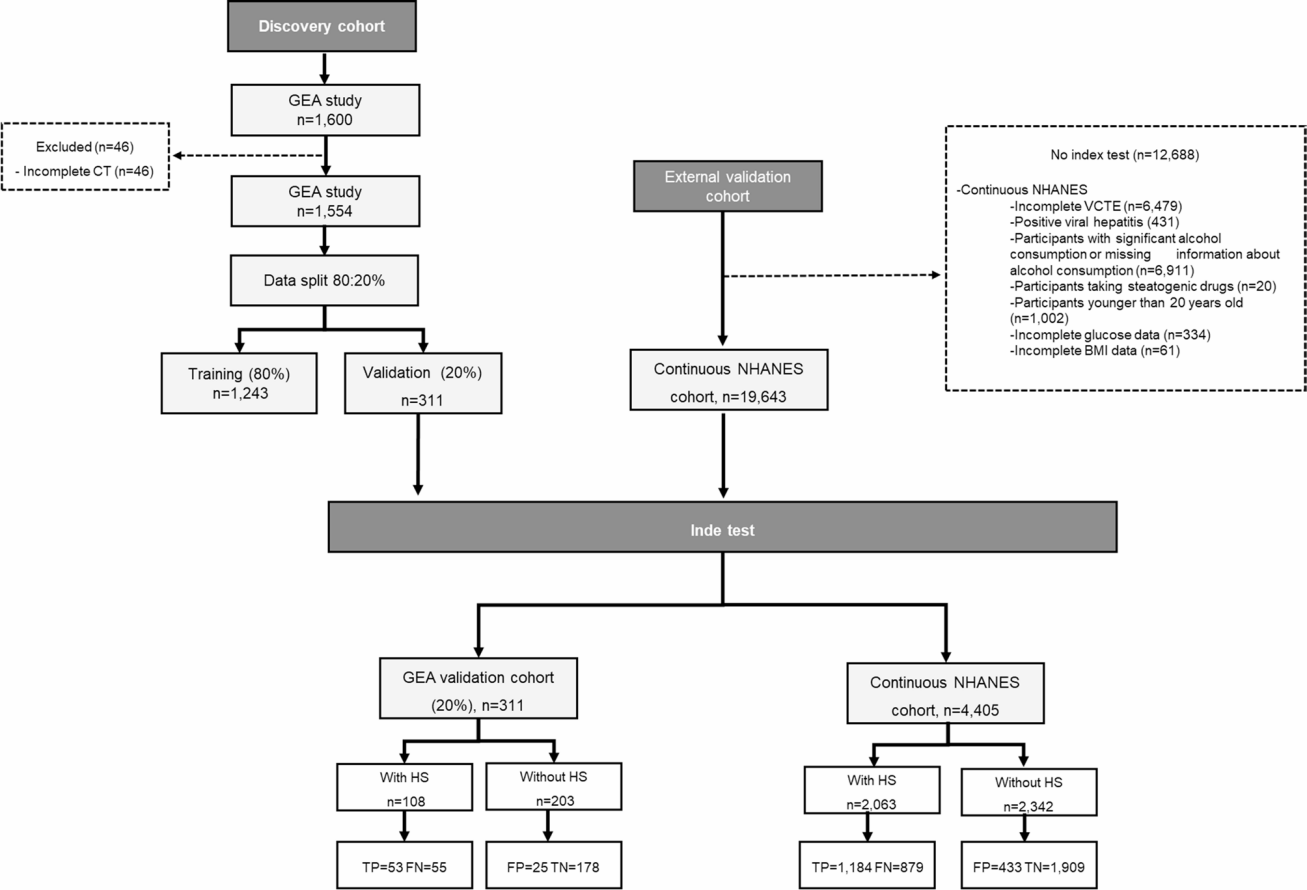
STARD diagram representing evaluated cohorts for development and validation of the model for identification of fatty liver (MOFI-FL) comparing across validation cohorts according to true and false positive and negative values. *Abbreviations*: TP: True positive, FP: False positive, TN: True negative, FN: False negative, MOFI-FL: model for identification of fatty liver, HS: hepatic steatosis, CT: computed tomography, VCTE: vibration-controlled transient elastography GEA: Genetics of Atherosclerotic Disease, *Continuous NHANES*: National Health and Nutrition Examination Survey 2017–2018 cycle.

In Fig. 2, we provide a detailed overview of the selection process from our studied cohorts, and in Table 1, we describe its characteristics. The characteristics of the GEA validation cohort (*n* = 311) were similar to the development sample. Conversely, external cohorts exhibit distinct clinical and biochemical characteristics. The *continuous NHANES* cohort had the highest percentage of men (56%) and was the oldest group (63 ± 14 years). When examining anthropometric measurements, the *continuous NHANES* cohort displayed a higher BMI (31 ± 7 kg/m^2^), WC (105 ± 16 cm), and prevalence of central obesity (65%). Additionally, the prevalence of T2D was higher in the *continuous NHANES* cohort at 39%, compared to just 32% in the GEA cohort (internal validation). Moreover, the GEA cohort showed a higher prevalence of MS (47%), whereas the *continuous NHANES* cohort had a lower proportion of this condition (13%). Notably, the prevalence of HS across the studied samples was 34% in the discovery sample, 35% in the GEA validation cohort, and 47% in the *continuous NHANES*.

**Table 1:** Clinical and biochemical characteristics of cohorts.

	GEA Cohort		Continuous NHANES cohort External validation data *n* = 4,405
	Training data *n* = 1,243	Internal validation data *n* = 311	
Sex, female n (%)	645 (52)	148 (48)	1,931 (44)
Age (years)	53 (9)	53 (9)	63 (14)
BMI (Kg/m^2^)	28 (4)	28 (4)	31 (7)
Overweight, n (%)	986 (79)	245 (78)	3428 (78)
Obesity, n (%)	394 (31)	99 (31)	2015 (46)
Waist Circumference (cm)	95 (12)	95 (11)	105 (16)
Central obesity, n (%)	596 (31)	144 (30)	2888 (65)
T2D, n (%)	411 (33)	100 (32)	1,730 (39)
Metabolic Syndrome (IDF-Criteria), n (%)	521 (42)	146 (47)	491 (13)
Hypertension, n (%)	396 (31)	92 (30)	2408 (46)
Glucose (mg/dL)	90 (84,99)	92 (86,100)	101 (91,125)
Insulin (µU/mL)	17 (13,24)	17 (13,24)	11 (7,18)
Uric acid (mg/dL)	5.61 (1.53)	5.69 (1.52)	5.60 (1.57)
Triglycerides (mg/dL)	147 (110,203)	164 (116,211)	154 (116)
Total cholesterol (mg/dL)	191 (167,213)	192 (169,215)	176 (148,203)
HDL-C (mg/dL)	44 (36,54)	43 (36,52)	49 (41,60)
LDL-C (mg/dL)	117 (97,137)	118 (98,138)	94 (73,118)
AST (U/L)	25 (21,30)	25 (20,32)	19 (15,23)
ALT (U/L)	24 (18,34)	24 (18,33)	16 (13,23)
GGT (U/L)	27 (18,42)	28 (20,46)	21 (15,31)
Hepatic steatosis n (%)	418 (34)	108 (35)	2,063 (47%)

Data is presented in mean (SD), n (%) and Median (IQR). BMI: body mass index, WC: waist circumference, T2D: type 2 diabetes, MS: metabolic syndrome, HDL-C: high-density lipoprotein cholesterol, LDL-C: low-density lipoprotein cholesterol, AST: aspartate aminotransferase, ALT: alanine aminotransferase, GGT: gamma-glutamyl transferase. T2D: fasting glucose level ≥ 126 mg/dL, the use of diabetes medications, or a previously reported diagnosis of diabetes. Overweight: BMI > 25 Kg/m^2^, Obesity: BMI > 30 Kg/m^2^. Central obesity: WC ≥ 102 cm for men and ≥ 88 cm for women. Hypertension: systolic blood pressure of ≥ 130 mmHg or a diastolic blood pressure of ≥ 85 mmHg. Metabolic syndrome (IDF-Criteria): if participants met at least three of the following criteria: High WC, triglycerides ≥ 150 mg/dL, low HDL cholesterol (< 40 mg/dL for men and < 50 mg/dL for women), hypertension, or fasting glucose ≥ 100 mg/dL. Hepatic steatosis was identified differently across validation cohorts. In the GEA study, it was defined by a liver-to-spleen attenuation ratio (L: S) of < 1.0 (CT). In NHANES cohorts, when controlled attenuation parameter (CAP) value > 285 dB/m.

.

### Inception of MOFI-FL

Using all possible combinations from our identified predictors, we selected the 10 best models based on AIC minimization in the discovery cohort, which included eight variables (insulin_log_, hs-CRP_log_, uric acid, glucose_log_, ALT_log_, GGT_log_, serum adiponectin_log_, and BMI_log_; **Supplementary Table 5**). Next, we developed a simplified approach based on the average importance of model terms (**Supplementary Fig. 2**). The resulted model included ALT_log_, glucose_log_, uric acid, and BMI_log_ and was termed *MOdel For Identification of Fatty Liver (MOFI-FL*) (**Supplementary Table 6**). The AUROC between the complete and simplified model was similar, with no discrepancies in systematic bias and a high correlation between models, assessed by the Bland-Altman and the intra-class correlation analyses, respectively (**Supplementary Fig. 3**). Our model explained 42% of the variability of HS (indicated by Nagelkerke’s pseudo-R^2^) and had an AUROC of 0.793 (95% CI: 0.76–0.82, **Supplementary Table 6**). The complete model assumptions, illustrated in **Supplementary Fig. 4**, demonstrate reliable performance. The observed and predicted data aligned well, the residuals were within the expected bounds, no influential observations were detected nor multicollinear, and the residuals met the normality assumptions. Additionally, the model’s reliable performance was further validated by cross-validation, which displayed good calibration and a net benefit in the decision curve analysis, as shown **in Supplementary Fig. 5**.

The resulting equation for MOFI-FL was defined as follows:

$$\begin{aligned} & \:MOFI-FL=\:-25.34+(1.56\times\:ln(ALT\:[U/L]\left)\:\right)+(0.18\times\:Uric\:acid\:[mg/dL\left]\right)\\&\qquad\qquad\qquad\quad+(1.11\times\:ln(Glucose\:[mg/dL]\left)\:\right)+(3.93\times\:ln(BMI\:[kg/{m}^{2}]\left)\:\right)\end{aligned}$$

With this equation, the predicted probability of HS could be derived as follows:

$$\:Probability\:of\:HS=\left(\frac{{exp}^{(MOFI-FL)}}{1+{exp}^{(MOFI-FL)}}\right)*100$$

In **Supplementary Table 7**, we present an example of a schematic case for its application and interpretation. To facilitate the application of MOFI-FL for clinical and research purposes, we developed a Shiny App, available at: https://juanreyesbarrera.shinyapps.io/MOFI-FL/.

### Internal validation and psychopathological correlations of MOFI-FL

The MOFI-FL index demonstrated adequate diagnostic performance in the internal validation cohort, which comprised 20% of the GEA study (*n* = 311). The AUROC of 0.78 (95% CI: 0.72–0.83) indicated an adequate ability to discriminate between individuals with and without HS with reasonable accuracy (75%, 95% CI: 69–79%, Table 2). The sensitivity was modest at 50% (95% CI: 39–58%), highlighting some limitations in detecting PPV, which was 68% (95% CI: 56–78%). In contrast, the specificity was high at 88% (95% CI: 82–91%) and the NPV was 76% (95% CI: 70–81%, Table 2). Additionally, MOFI-FL exhibited clear associations with metabolic parameters. Higher predicted probabilities from the MOFI-FL index strongly correlated with elevated fasting insulin (ρ = 0.41, 95% CI: 0.32 to 0.50) and increased visceral fat area (ρ = 0.57, 95% CI: 0.48 to 0.63), supporting its relevance in assessing metabolic risk. Conversely, there was a negative correlation with serum adiponectin (ρ= -0.22, 95% CI: -0.33 to -0.12, **Supplementary Fig. 6**).

**Table 2:** Performance of MOFI-FL and others hepatic steatosis indices.

*Cohort*	*Model*	Discrimination								Explained variation	
		*Accuracy %*	*Sensitivity%*	*Specificity%*	*PPV%*	*NPV%*	*LR+*	*LR-*	*AUROC*	*Deviance*	*BIC*
*GEA internal validation cohort*	**MOFI-FL**	**74 (69**,**79)**	**50 (39**,**58)**	**88 (82**,**91)**	**68 (56**,**78)**	**76 (70**,**81)**	**3.9 (3.0**,**5.0)**	**0.59 (0.45**,**0.77)**	**0.78 (0.72**,**0.83)**	**323**	**335**
	FLI	70 (64,74)	45 (34,54)	81 (76,87)	56 (46,68)	73 (67,79)	2.5 (2.0,3.3)	0.67 (0.52,0.86)	0.70 (0.65,0.77)	347	358
	HSI	67 (61,72)	32 (21,39)	86 (81,91)	56 (41,68)	70 (63,75)	2.3 (1.7,3.2)	0.81 (0.64,1.01)	0.72(0.66,0.78)	360	371
	NAFLD-LFS	69 (63,74)	32 (23,42)	89 (83,92)	61 (46,72)	71 (65,76)	2.8 (2.1,3.8)	0.76 (0.60,0.96)	0.74(0.69, 0.80)	358	369
	AST/ALT	65 (64,74)	17 (10,25)	98 (95,99)	81 (59,94)	68 (63,74)	0.2(0.1,0.3)	0.02 (0.007,0.005)	0.67(0.61,0.74)	393	404
Continuous NHANES	**MOFI-FL**	**70** (68,71)	**73** (71,75)	68 (66,70)	57 (55,59)	**81**(79,83)	**2.3 (2.2**,**2.3)**	**0.40 (0.36**,**0.40)**	**0.77 (0.76**,**0.78)**	**5179**	**5195**
	FLI	71 (68,71)	74 (72,76)	69 (67,71)	68 (67,71)	74 (72,76)	2.3 (2.3,2.4)	0.39 (0.36,0.40)	0.79 (0.79,0.81)	4494	4511
	HSI	69 (67,70)	61 (58,63)	76 (74,77)	75 (74,77)	68 (67,70)	2.5 (2.4,2.5)	0.51(0.50,0.55)	0.77 (0.76,0.79)	5106	5123
	NAFLD-LFS	71 (69,72)	61 (59,92)	80 (78,81)	74 (73,75)	68 (67,69)	3.0 (3.1,4.1)	0.49 (0.10,0.51)	0.78 (0.76,0.80)	2278	2293
	AST/ALT	61 (59,62)	53 (48,60)	68 (63,72)	60 (58,61)	62 (61,67)	1.7 (1.6,1.7)	0.69 (0.63.0.72)	0.62 (0.62,0.65)	5847	5864

Values in parentheses represent the 95% confidence interval (CI). AUROC: Area Under the Receiver Operator Curve, FLI: fatty liver index, HSI: hepatic steatosis index, NAFLD-LFS: non-alcoholic fatty liver disease liver fat score, AST/ALT: aspartate aminotransferase/alanine aminotransferase ratio, MOFI-FL: model of identification of fatty liver, PPV: positive predictive value, NPV: negative predictive value, LR+: positive likelihood ratio, LR-: negative likelihood ratio, AUC: area under the roc curve.

### External validation of MOFI-FL against VCTE

Across validation cohorts, MOFI-FL performed adequately in HS identification. Accuracy trended the same and varied slightly according to validation cohorts. Validated against VCTE in the *continuous NHANES* cohort, MOFI-FL exhibited moderate performance (AUROC: 0.77, 95% CI: 0.76–0.78) and accuracy (70%, 95% CI: 68–71). In this dataset, sensitivity improved to 73% (95% CI: 71–75%), while specificity remained at 68% (95% CI: 66–70%). The complete performance and diagnostic metrics for MOFI-FL are presented in Table 2. We also assessed the performance and clinical utility of the MOFI-FL index and its individual components across cohorts. The results of this additional analysis indicate that the MOFI-FL index consistently demonstrated both clinical utility and good performance compared to its individual components (**Supplementary Table 8 and Supplementary Fig. 7**).

### Comparison of MOFI-FL with other HS indexes

The MOFI-FL index demonstrated competitive performance compared to established HS indices, including FLI, HSI, NAFLD-LFS, and AST/ALT, across all cohorts. In the GEA validation cohort, MOFI-FL achieved a slightly higher AUROC (0.78, 95%CI: 0.72–0.83,) than these indices, whose AUROCs were as follows: FLI (0.70, 95% CI: 0.65–0.77), HSI (0.72, 95% CI: 0.66–0.78), NAFLD-LFS (0.74, 95% CI: 0.69–0.80), and AST/ALT (0.67, 95% CI: 0.61–0.74) (**Supplementary Fig. 8A**). However, the differences in AUROC values were modest, with NAFLD-LFS and HSI also demonstrating similar performance. When examining the cohort assessed with VCTE, *continuous NHANES* cohort, the AUROCs of these surrogates were generally comparable (**Supplementary Fig. 8B)**. Except for the AST/ALT ratio, MOFI-FL and the other indices showed similarly robust discrimination for HS detection. Detailed performance metrics and AUROC comparisons can be found in Table 2. Additionally, we assessed the clinical benefit of MOFI-FL based on decision curves. These analyses indicate that MOFI-FL consistently demonstrates a positive net benefit across cohorts, especially at low to moderate risk thresholds (**Supplementary Fig. 9**).

### Prediction of all-cause and cause-specific mortality using MOFI-FL

**Fig. 3:**
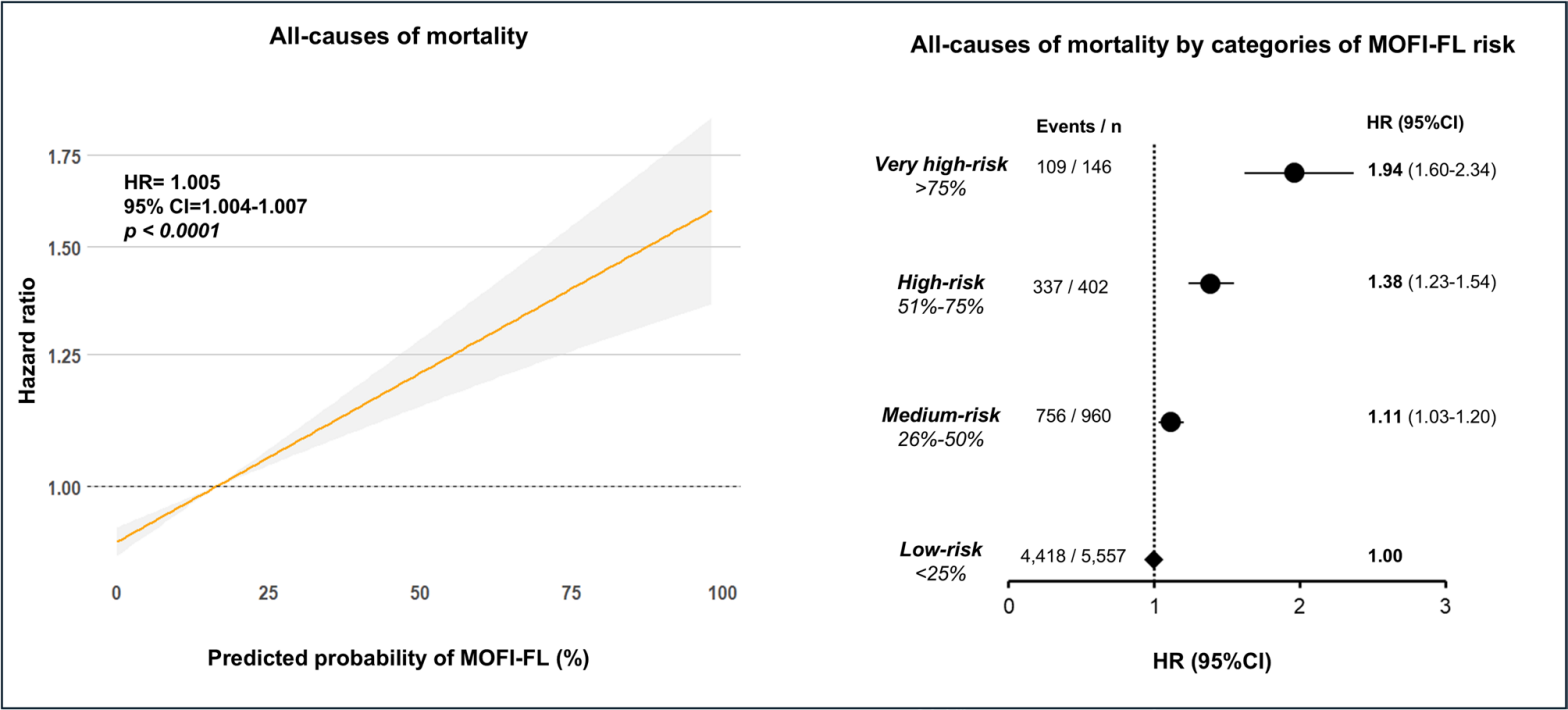
Risk prediction for all-cause mortality using MOFI-FL. The left panel illustrates the association between MOFI-FL predicted probabilities (as a continuous variable) and the relative risk (hazard ratio, HR) for all-cause mortality, adjusted for age and sex. The right panel presents risk predictions categorized into low, medium, high, and very high risk MOFI-FL predicted probabilities, adjusted for age and sex. Both models were adjusted for age and sex in all analyses.

The initial dataset included 23,910 participants from NHANES-III. We excluded 10,044 subjects under 20 years of age, 85 subjects due to missing mortality records, and 1,097 subjects due to missing data to estimate MOFI-FL. Consequently, data from 12,684 participants were analyzed to evaluate whether MOFI-FL could predict all-cause and cause-specific mortality. Descriptive characteristics of these participants are provided in **Supplementary Table 9**. The mean age was 49 (± 20) years and women constituted 53% of the sample. The ethnic distribution included 43% Caucasians, 26% Afro-Americans, 26% Mexican Americans, and 4% from other ethnicities. During a median follow-up of 317 (IQR: 178–347) person-months, 5,620 participants (44%) died from any cause. Cardiovascular disease was the most frequent cause of death (14%), followed by diabetes-related (1.5%) and nephrological (0.7%) deaths. Using age- and sex-adjusted Cox proportional hazards models, we observed a positive association between a 1% increase in MOFI-FL and all-cause mortality (HR = 1.005, 95% CI: 1.004–1.007). Furthermore, when we stratified participants into low-, medium-, high-, and very high-probability groups, we noted a progressive increase in the risk of all-cause mortality. Notably, those in the very high-probability category had a nearly two-fold (HR = 1.94, 95% CI: 1.60–2.34) increased risk of all-cause mortality compared with the low-probability group (Fig. 3). Cause-specific mortality analyses revealed that a 1% increase in MOFI-FL probability positively correlated with higher risk of cardiovascular (HR = 1.008, 95% CI: 1.005–1.010), diabetes-related (HR = 1.034, 95% CI: 1.028–1.040), and nephrological (HR = 1.012, 95% CI: 1.000–1.024) deaths. Moreover, we observed an increasing risk across medium-, high-, and very high-probability groups for both cardiovascular and diabetes-related mortality. Specifically, participants in the very high-probability category experienced a 1.7-fold increased risk (95% CI: 1.40–2.53) for cardiovascular deaths and an 8.60-fold increased risk (95% CI: 4.67–15.8) for diabetes-related deaths. In contrast, we found no significant association between MOFI-FL probabilities and mortality from chronic lower respiratory tract diseases, cerebrovascular diseases, or malignant neoplasms. As a sensitivity analysis, all previous models were further adjusted for ethnicity and the number of comorbidities (Table 3). Overall, the same trends were observed after this adjustment. The relationship between the MOFI-FL and mortality, both from all causes and specific causes, remained consistent, although some associations remain marginal. Finally, in a secondary analysis, we evaluated the relative prognostic performance of MOFI-FL by comparing it with other established hepatic steatosis indices (FLI, HSI, NAFLD-LFS, and the AST/ALT ratio) using Cox regression models and the concordance index (C-index). This analysis included only participants with complete data for all indices (*n* = 8,562). Although indices such as FLI showed slightly higher hazard ratios for some mortality outcomes (Supplementary Table 10), MOFI-FL demonstrated comparable discriminatory performance for all-cause mortality. Despite showing the lowest univariate C-index (0.505), MOFI-FL achieved a final C-index of 0.845 after full adjustment for age, sex, ethnicity, and comorbidities—similar to values observed for FLI, HSI, and NAFLD-LFS (Supplementary Table 11).

**Table 3:** Risk prediction for specific causes of mortality by MOFI-FL predicted probabilities.

Mortality cause	Predicted probability of MOFI-FL *n* = 12,684^1^	Medium risk 26–50, *n* = 1,716^1^	High risk 51–75, *n* = 739^1^	Very high risk > 75, *n* = 254^1^
*Cardiovascular*				
Model 1	1.008 (1.005,1.010) **	1.10 (0.95,1.27)	1.70 (1.40,2.06) **	1.70 (1.14,2.53) *
Model 2	1.004 (1.001,1.007) **	0.98 (0.85,1.14)	1.44 (1.18,1.75) **	1.39 (0.93,2.08)
*Diabetes*				
Model 1	1.034 (1.028,1.040) **	2.91 (2.04,4.14) **	4.99 (3.27,7.59) **	8.60 (4.67,15.8) **
Model 2	1.026 (1.020,1.033) **	2.15 (1.51,3.07) **	3.38 (2.20,5.17) **	5.38 (2.90,9.99) **
*Nephrological diseases*				
Model 1	1.012 (1.000,1.024) ^†^	1.05 (0.55,2.01)	1.64 (0.70,3.81)	2.50 (0.60,10.3)
Model 2	1.008 (0.991,1.010)	1.92 (0.48,1.77)	1.44 (0.61,3.39)	2.08 (0.50,8.65)
*Cerebrovascular diseases*				
Model 1	1.006 (0.999,1.012)	1.20 (0.83,1.62)	1.34 (0.84,2.14)	1.32 (0.49,3.56)
Model 2	1.004 (0.997,1.010)	1.09 (0.81–1.49)	1.25 (0.78,2.01)	1.20 (0.44,3.26)
*Malignant neoplasms*				
Model 1	0.997 (0.994,1.001)	1.07 (0.91,1.25)	0.81 (0.61,1.07)	1.03 (0.62,1.69)
Model 2	0.997 (0.994,1.001)	1.03 (0.88,1.21)	0.78 (0.59,1.05)	1.01 (0.61,1.66)
*Chronic lower respiratory diseases*				
Model 1	0.991 (0.98,1.000)	0.70 (0.45,1.09)	1.06 (0.59,1.92)	0.78 (0.19,3.15)
Model 2	0.988 (0.97,0.997)	0.65 (0.42,1.02)	0.92 (0.50,1.16)	0.65 (0.16,2.66)

Model 1: adjusted by age and sex.

Model 2: Model 1 plus ethnicity and number of comorbidities.

^*1*^ HR = Hazard ratio, 95% CI = Confidence interval.

†*p* < 0.05* *p* < 0.005, **p < 0.0001.

## Discussion

In this study, we developed MOFI-FL, a novel clinical surrogate for detecting HS. We then validated this index against VCTE, demonstrating general good diagnostic performance, thus confirming its validity and generalizability. The performance of MOFI-FL was comparable to four existing HS indices, and notably, it also predicted all-cause mortality, especially cardiometabolic causes. Overall, MOFI-FL showed adequate accuracy in identifying adults with HS, suggesting its potential as a practical and accessible tool for both clinical use and epidemiological research.

As liver biopsy remains limited by its invasiveness and specialized requirements, image-based and clinical surrogates have become increasingly important for detecting HS in epidemiological and clinical settings^38^. This is especially relevant in LMICs, where environmental and lifestyle factors compound the already high burden of HS^39^. Therefore, adopting non-invasive, accurate, and cost-effective strategies to evaluate HS is critical for facilitating earlier detection and more efficient intervention to prevent related complications^40,41^. In our study, we developed a simple index that demonstrated reliable performance in identifying individuals with HS, which we externally validated against VCTE. The relevance of MOFI-FL as a tool for detecting HS is supported by its overall performance and its pathophysiological correlation within three key cardiometabolic markers involved in the pathophysiology of ectopic fat accumulation in the liver. First, MOFI-FL was inversely correlated with adiponectin, reflecting impaired subcutaneous adipose tissue accumulation and insulin resistance^42^. Second, it was positively correlated with fasting insulin, a well-recognized marker of glycemic dysregulation^43^. Finally, MOFI-FL also positively correlated with increased visceral adipose tissue, which has been linked to steatohepatitis and artery calcification in patients with HS^44,45^. Collectively, these correlations suggest that MOFI-FL captures underlying mechanisms of ectopic fat deposition, thereby potentially identifying individuals at risk of T2D, MS, and kidney dysfunction. This pathophysiological basis further underscores MOFI-FL’s utility in clinical practice, where a simple, accessible index can aid in early identification and risk stratification for HS and its related comorbidities.

MOFI-FL was developed with a strong emphasis on practical and simplified applications. Unlike other indices, it does not depend on costly biochemical tests, imaging studies, or prior diagnostic constructs. In contrast, alternative indices such as the NAFLD-LFS necessitate two prior diagnoses—MS and T2D—requiring multiple measurements or previous evaluations, as well as non-routine tests such as glycated hemoglobin, which may not be readily accessible in LMICs settings^35^; similarly, the HSI also requires prior T2D diagnoses^34^. Moreover, AST/ALT ratio and the FLI only require clinical and biochemical parameters, but existing studies indicate that these indices exhibit modest efficacy in detecting HS^46^. While AST/ALT was originally used to assess liver fibrosis, it is often included in discussions about steatosis because it is commonly used as a proxy for this condition. When considering the FLI, two of its key components (WC and GGT) could have some limitations. WC can be affected by inter-observer variability, which reduces its reliability in both clinical and research environments^47–49^. Similarly, GGT lacks specificity; elevated levels may indicate not only steatosis but also factors such as alcohol consumption, medication use, or systemic inflammation^14,50^. These issues may undermine the accuracy of the FLI in certain contexts. Furthermore, a comprehensive population-based study revealed that NAFLD-LFS, HSI, and FLI provide diagnostic efficacy ranging from 70 to 80%, accompanied by lower sensitivities and specificities than originally described^51^. Although these indices have undergone independent validation, comparing their diagnostic performances remains challenging due to differences in patient populations and validation methods and their underperformance in individuals with comorbid conditions^52,53^. These limitations not only make MOFI-FL an attractive and cost-effective strategy to detect HS but also surpass some technical limitations of other indexes.

HS is associated with increased mortality, largely driven by adverse cardiometabolic profiles^54^. In our study, we found that MOFI-FL was associated with all-cause mortality, particularly deaths from cardiometabolic causes (e.g., diabetes-, cardiovascular- and nephrotic-related deaths). This association was stronger among individuals whose MOFI-FL predicted probabilities exceeded > 75%. The relationship between MOFI-FL and increased mortality likely stems from the model’s components. Despite its simplicity, MOFI-FL’s components cover key aspects of HS pathophysiology, such as increased adiposity (BMI), glucose dysregulation, impaired liver function (ALT), and altered protein metabolism (uric acid), all of which have been linked with higher mortality risk^55–58^. Nevertheless, the index outperforms all individual components separately, indicating that its combination likely captures mortality risk related to the synergistic occurrence captured by its individual components. In comparative analyses of mortality prediction, MOFI-FL demonstrated a discriminatory performance comparable to that of traditional HS indices, with similar C-index values across models. Notably, although its univariate discriminatory ability was limited, MOFI-FL showed an increase in predictive capacity after adjusting for demographic and clinical covariates. Overall, these results highlight MOFI-FL’s potential clinical value for identifying individuals at higher risk of death, emphasizing the need for further research to explore its application in risk stratification and targeted interventions for HS. Nevertheless, incorporating these scoring systems into routine clinical practice remains under debate and has been constrained by limitations in their diagnostic efficacy. Further research is imperative to validate the performance of MOFI-FL, as well as other indexes to clarify their role in clinical decision-making.

### Strengths and limitations

Our study offers strengths that should be highlighted. MOFI-FL was modeled using CT as our gold standard, which remains a strong and accessible method to detect HS within epidemiological studies. Moreover, MOFI-FL was also validated against VCTE, and it not only identified HS but also predicted all-cause and cardiometabolic mortality. Despite these strengths, several limitations should be acknowledged. First, additional evaluations in diverse populations, including healthy individuals and those with various comorbidities, are needed to confirm MOFI-FL’s validity in clinical practice. Second, we were unable to validate MOFI-FL against other commercially available serum markers, such as SteatoTest. Third, while the MOFI-FL performed comparably to existing indices, in some cases, traditional performance metrics for other indices might have been slightly better. However, our model showed distinct advantages in DCA and an NPV, which could support its potential utility as a complementary tool in specific clinical settings. Additionally, we acknowledge that our model may not outperform machine learning-based models; however, our primary goal was to develop a parsimonious and easy-to-implement tool. Importantly, some studies suggest that simpler models, even if less accurate, maybe more practical and scalable in real-world scenarios, providing a reassuring perspective on our approach^59^. Fourth, age and sex were not directly included in the MOFI-FL because their effects on HS risk are likely captured indirectly through metabolic biomarkers in the index^60–62^. This approach maintains the simplicity and balance of the score, striking a balance between accuracy and ease of use. Fifth, this study’s limitation is that liver biopsies, considered the gold standard for diagnosing and assessing HS, were not feasible. However, liver biopsies are invasive, carry potential risks, and may not be representative of the entire liver due to sampling variability. Finally, relying solely on the traditional definition of HS may be limiting. The terminology in this field has recently evolved, with metabolic dysfunction–associated steatotic liver disease (MASLD) now accepted as the preferred term, as it better reflects the metabolic dysfunction underlying fatty liver disease. While MOFI-FL was designed to detect liver fat accumulation, we acknowledge that it does not capture the full metabolic criteria required for MASLD diagnosis^63^. Nonetheless, MOFI-FL could be implemented as a simple, non-invasive, and surrogate of liver fat accumulation within the MASLD diagnostic framework. Current MASLD definitions require imaging or biopsy evidence of hepatic steatosis, which can be problematic in low-resource settings. Future studies should evaluate the integration of MOFI-FL into the MASLD definition, potentially complementing or substituting imaging and biopsy requirements to enhance early detection of MASLD and improve risk stratification, thereby refining their clinical applicability.

## Conclusion

In conclusion, MOFI-FL is a novel and simple index that utilizes accessible laboratory and anthropometric metrics to detect HS. It achieves performance comparable to previously validated indices and predicts both all-cause and cardiometabolic mortality. Overall, MOFI-FL offers a reliable approach that can be implemented in primary care and a variety of healthcare and epidemiological settings. As definitions and nomenclature for fatty liver disease continue to evolve, MOFI-FL may further facilitate the integration of emerging classifications (such as MAFLD and MASLD) into routine practice—ultimately enhancing both the prevention and management of cardiometabolic complications and beyond.

## Supplementary Information

Below is the link to the electronic supplementary material.

Supplementary Material 1

## Data Availability

All code, datasets, and materials necessary for the reproducibility of results are available at: https://github.com/REYJUA12235/MOFI-FL-manuscript. GEA dataset: To access the GEA study data, a formal request must be submitted to the corresponding researcher and to Rosalinda Posadas-Sanchez (rossy_posadas_s@yahoo.it). NHANES datasets: Publicly available NHANES datasets can be downloaded from: https://wwwn.cdc.gov/nchs/nhanes/continuousnhanes/default.aspx.  Mortality data: Linked mortality data from NHANES can be accessed at: https://www.cdc.gov/nchs/data-linkage/mortality-public.html.
