# Supplementary material for: MOFI-FL, a novel score for detecting hepatic steatosis and predicting cardiometabolic mortality
Source: Sci Rep. 2025 Aug 19;15:30363. doi: 10.1038/s41598-025-15487-7 (PMC12365217; doi:10.1038/s41598-025-15487-7)
Supplement: Supplementary file 1 — Supplementary Material 1 [file 41598_2025_15487_MOESM1_ESM.docx]

**SUPPLEMENTARY MATERIAL**

**MOFI-FL, a novel score for detecting hepatic steatosis and predicting cardiometabolic mortality**

Juan Reyes-Barrera, Rosalinda Posadas-Sánchez, Gilberto Vargas-Alarcón, Guillermo Cardoso-Saldaña, Paloma Almeda-Valdes, Omar Yaxmehen Bello Chavolla, Luis Ortiz-Hernández, and Neftali Eduardo Antonio-Villa.

Supplementary Figure 1__________________________________________________________2

Supplementary Figure 2__________________________________________________________3

Supplementary Figure 3__________________________________________________________4

Supplementary Figure 4__________________________________________________________5

Supplementary Figure 5__________________________________________________________6

Supplementary Figure 6__________________________________________________________7

Supplementary Figure 7 __________________________________________________________8

Supplementary Figure 8 __________________________________________________________9

Supplementary Figure 9 _________________________________________________________10

Supplementary Table 1________________________________________________________11-14

Supplementary Table 2________________________________________________________15-16

Supplementary Table 3 _______________________________________________________17-19

Supplementary Table 4 _________________________________________________________ 20

Supplementary Table 5 _________________________________________________________21

Supplementary Table 6 _________________________________________________________22

Supplementary Table 7 _______________________________________________________23-24

Supplementary Table 8 _________________________________________________________ 25

Supplementary Table 9 _________________________________________________________ 26

Supplementary Table 10________________________________________________________ 27

Supplementary Table 11 ________________________________________________________ 28

REFERENCES________________________________________________________________27


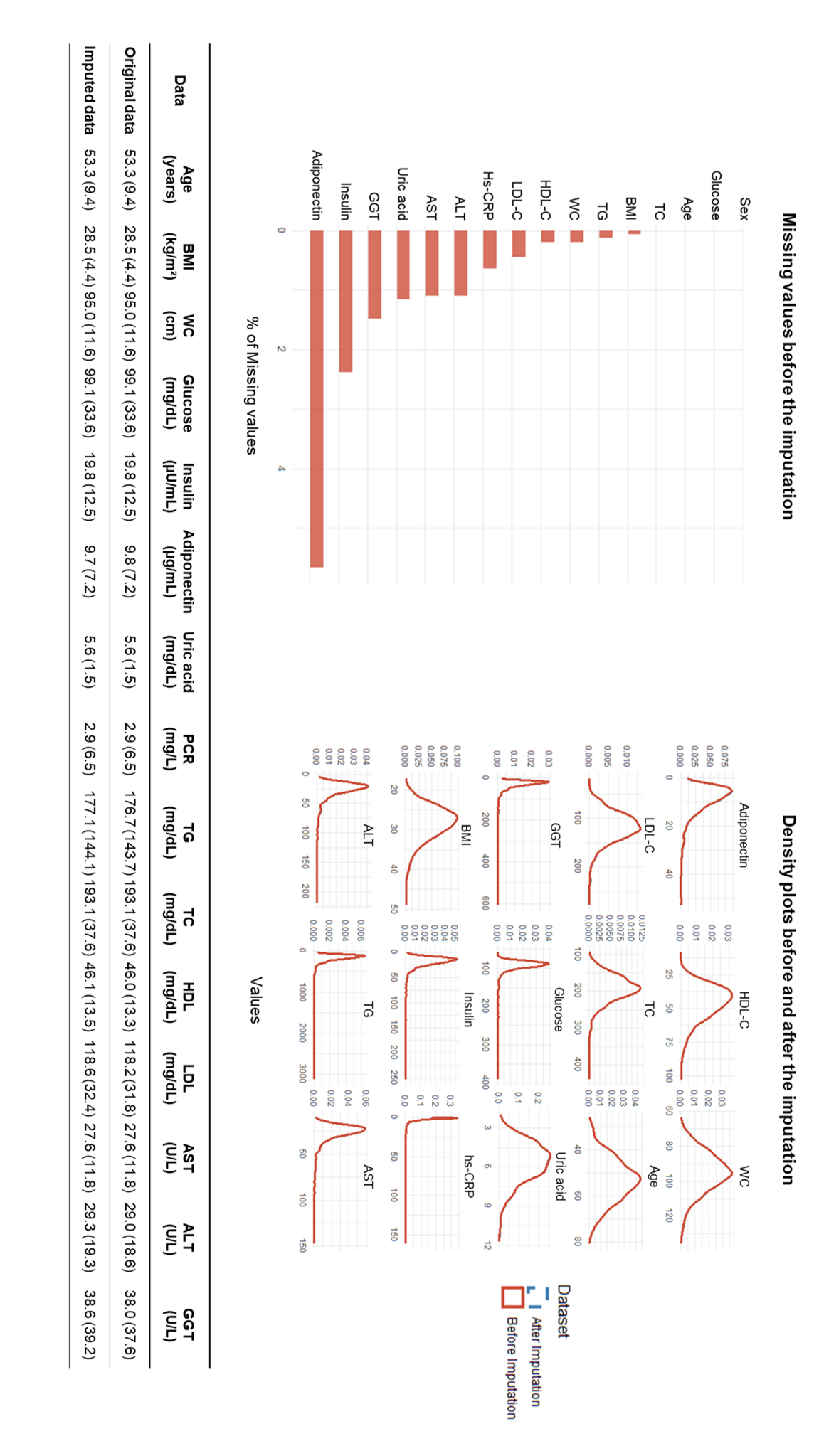


**Supplementary Figure 1.** Evaluation of missing data and imputation effect on distribution of variables. Left Panel: A bar plot shows the percentage of missing data for each variable in the dataset. Right Panel: Density plots compare the distributions of the variables before and after data imputation. The red line represents the distribution before imputation, and the blue dashed line indicates the distribution after imputation. Bottom Panel: A summary table provides an overview of the missing data for each variable after and before imputation. Values are presented as means (SD). BMI: Body mass index. WC: Waist circumference. hs-CRP: C-reactive protein. TG: triglycerides. TC: total cholesterol. HDL: high-density lipoprotein cholesterol. LDL: low-density lipoprotein cholesterol. AST: aspartate aminotransferase. ALT: alanine aminotransferase. GGT: gamma-glutamyl transferase

**Supplementary Figure 2.** Average importance of model terms for predicting HS. hs-CRP: high-sensitivity c-reactive protein, ALT (alanine aminotransferase), GGT (gamma-glutamyl transferase), BMI: body mass index, WC: waist circumference, LDL-C: low-density lipoprotein cholesterol, TC: total cholesterol, HDL-C: high-density lipoprotein cholesterol.


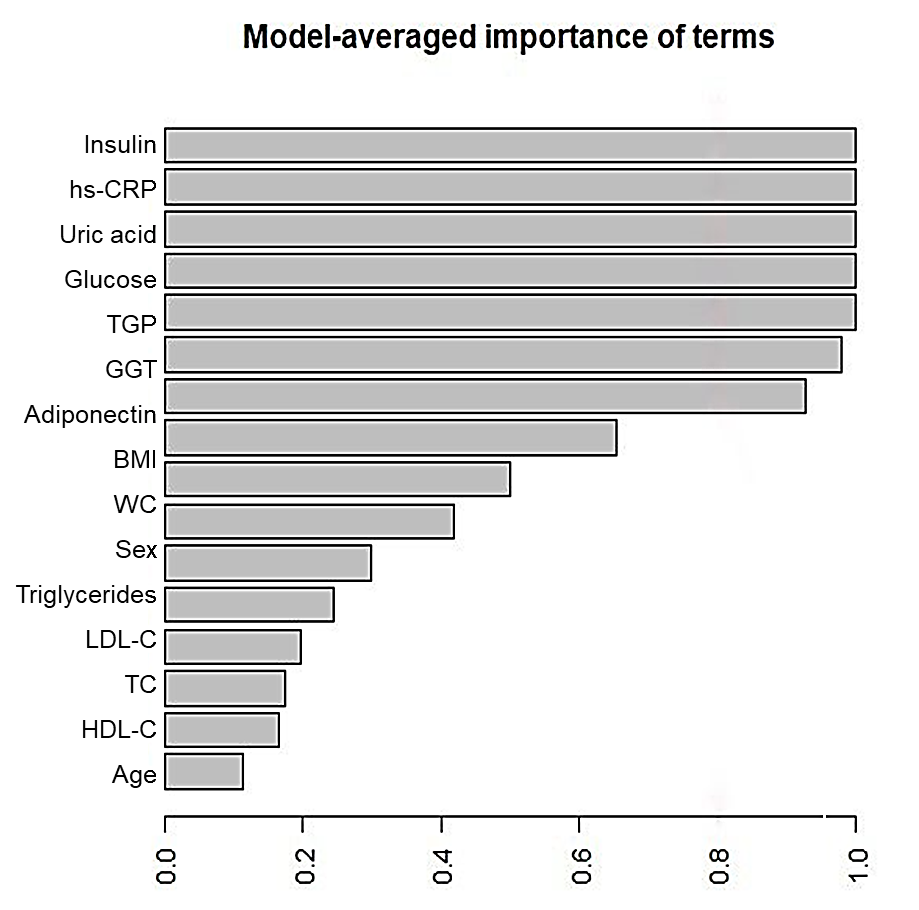


**Supplementary Figure 3.** Comparative analysis of models complete and simplified. Panel (a) shows the ROC curve, comparing the performance of the complete and simplified models. Panel (b) presents the Bland-Altman comparison to evaluate agreement between the models. Panel (c) illustrates the interclass correlation coefficient (ICC) to assess consistency between the models.


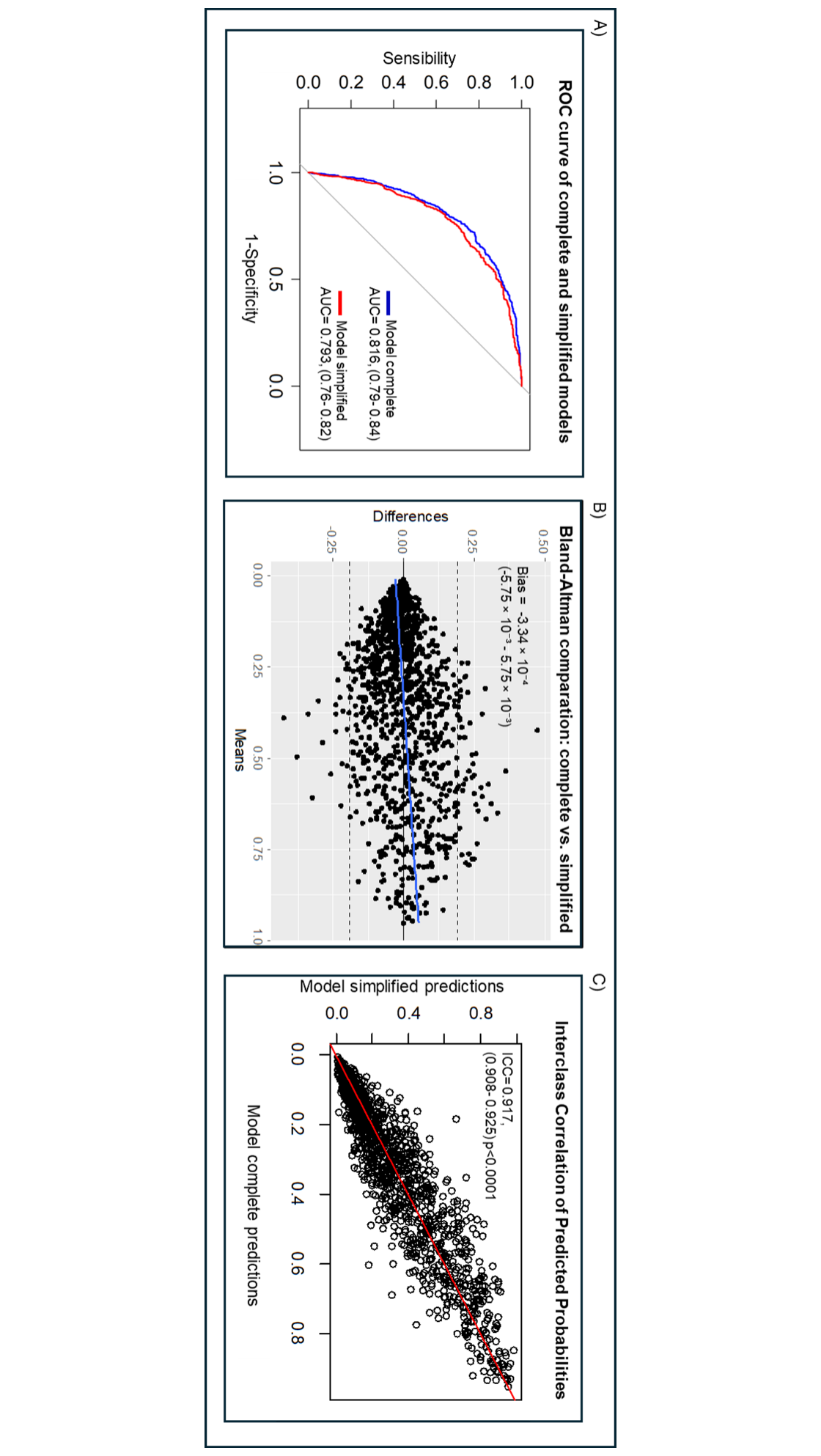


**Supplementary Figure 4.** MOFI-FL model assumptions check: Diagnostic plots evaluating the predictive model for HS. (Top left) Posterior predictive check showing observed and model-predicted data. (Top right) Binned residuals analysis, where points within error bounds indicate a good fit. (Middle left) Influential observations assessed using standardized residuals vs. leverage. (Middle right) Collinearity diagnostics using the variance inflation factor (VIF), indicating acceptable levels (VIF < 5). (Bottom) Uniformity of residuals assessed by quantile-quantile (Q-Q) plot against the standard uniform distribution. BMI: body mass index, ALT: alanine aminotransferase.


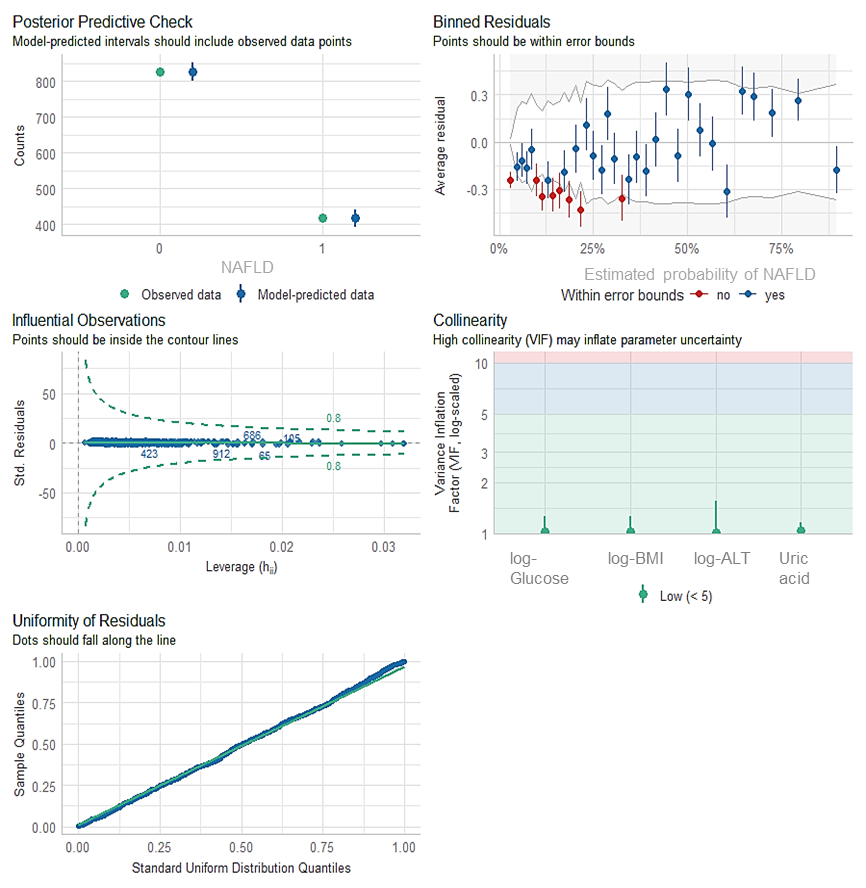


**Supplementary Figure 5.** MOFI-FL evaluation in training data. Model evaluation for predicting HS disease using the MOFI-FL model in the training dataset. (Top left) AUC values for each fold in a 10-fold cross-validation. (Top right) The calibration curve assesses agreement between predicted probabilities and observed outcomes, including metrics like the Hosmer-Lemeshow test and Brier score. (Bottom left) ROC curve with an AUC of 0.793 (95% CI: 0.76–0.82) shows the trade-off between sensitivity and specificity. (Bottom right) Decision curve analysis illustrates the MOFI-FL model's net benefit compared to classifying all or none as high-risk.


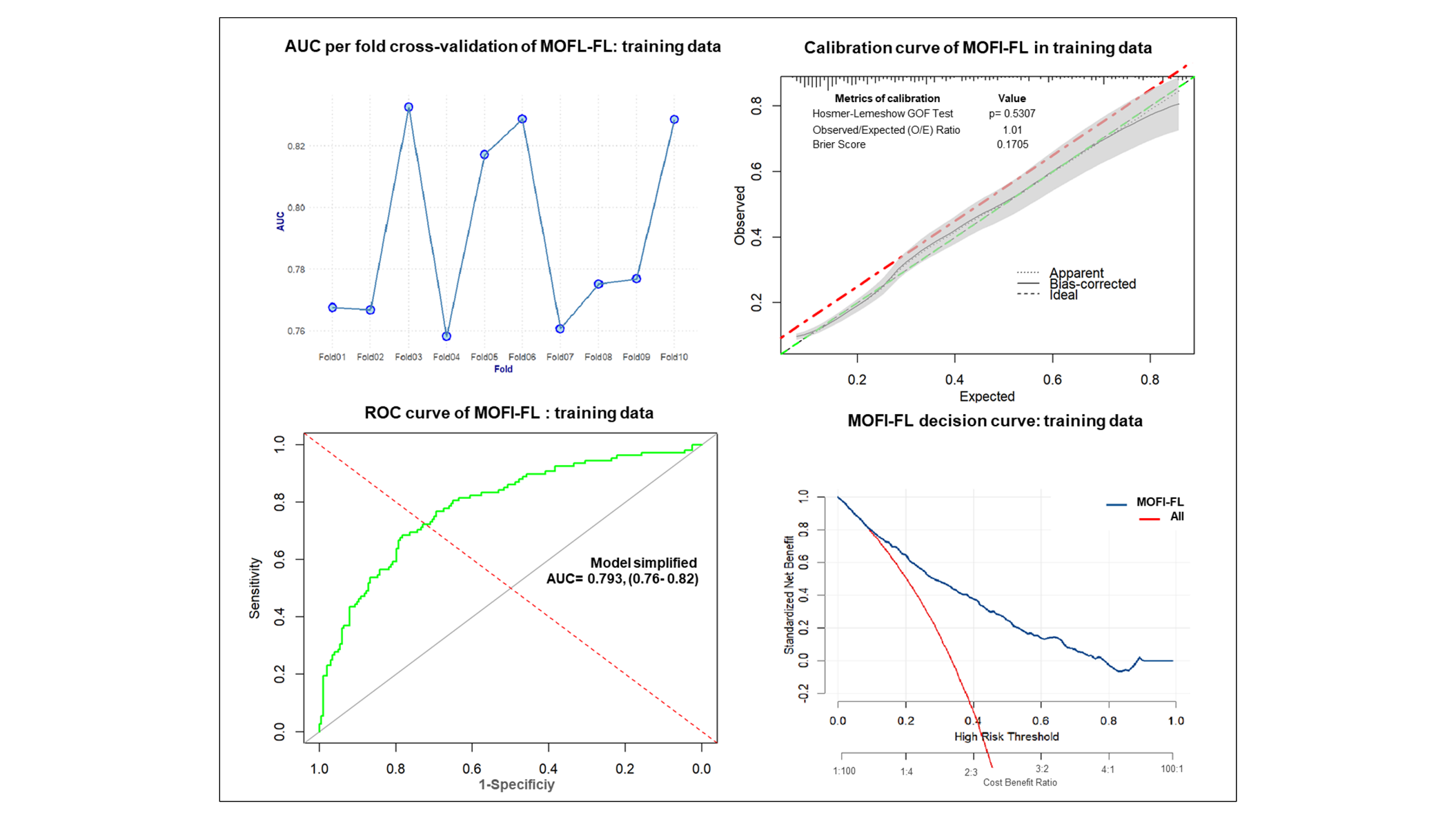


**Supplementary figure 6.** Correlation of predicted probabilities of MOFI-FL with insulin, adiponectin and visceral fat area in GEA internal validation data (n=311). CT: computed tomography.


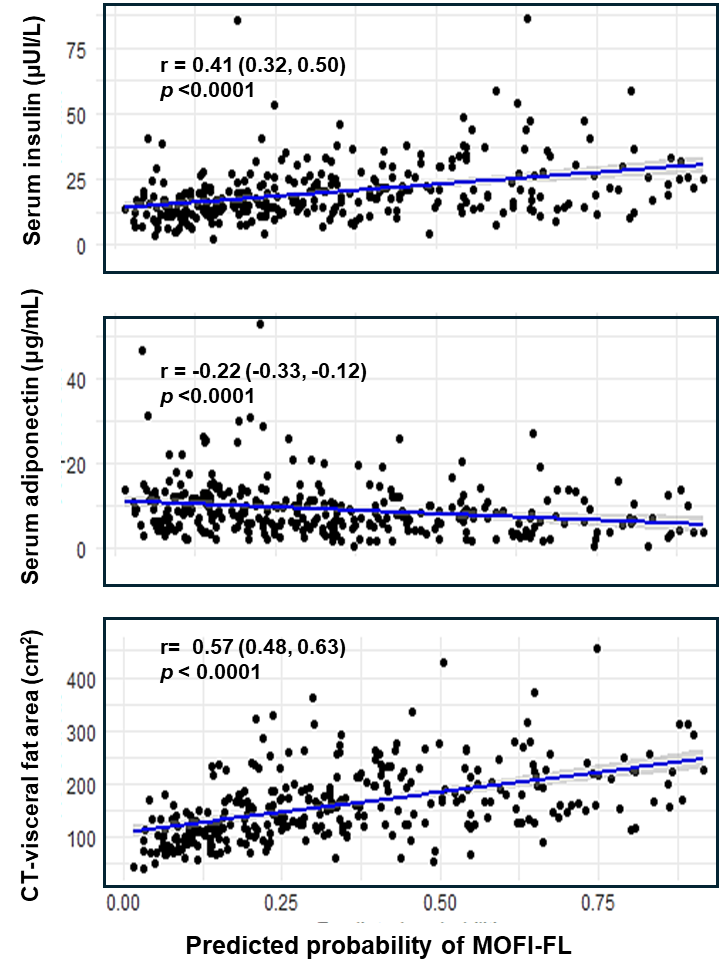


### **Supplementary figure 7.** Decision curve analysis comparing the net benefit of MOFI-FL and its components for HS identification.

### **
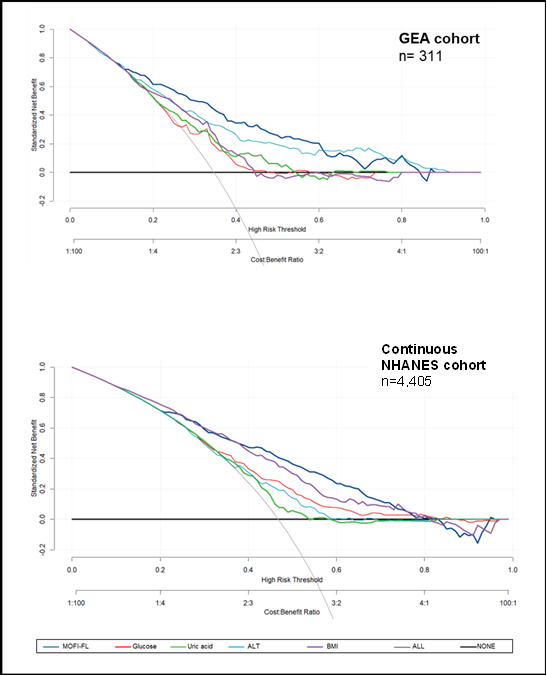
**

**Supplementary figure 8.** Areas under the receive operating characteristic (ROC) curves to detect HS across cohorts. FLI: fatty liver index, HSI: hepatic steatosis index, NAFLD-LFS: non-alcoholic fatty liver disease liver fat score, AST/ALT: aspartate aminotransferase/alanine aminotransferase ratio, MOFI-FL: model for identification of fatty liver, PPV: positive predictive value, NPV: negative predictive value, AUC: area under the curve.

###
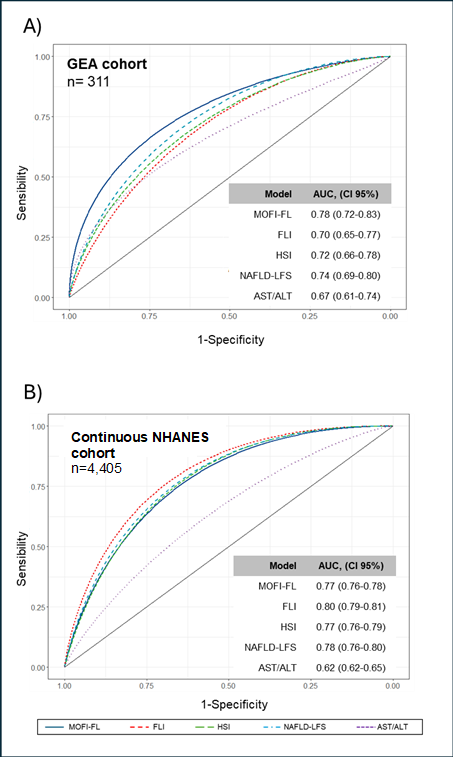


### **Supplementary figure 9.** Decision curve analysis comparing the net benefit of MOFI-FL with other indices for HS identification.


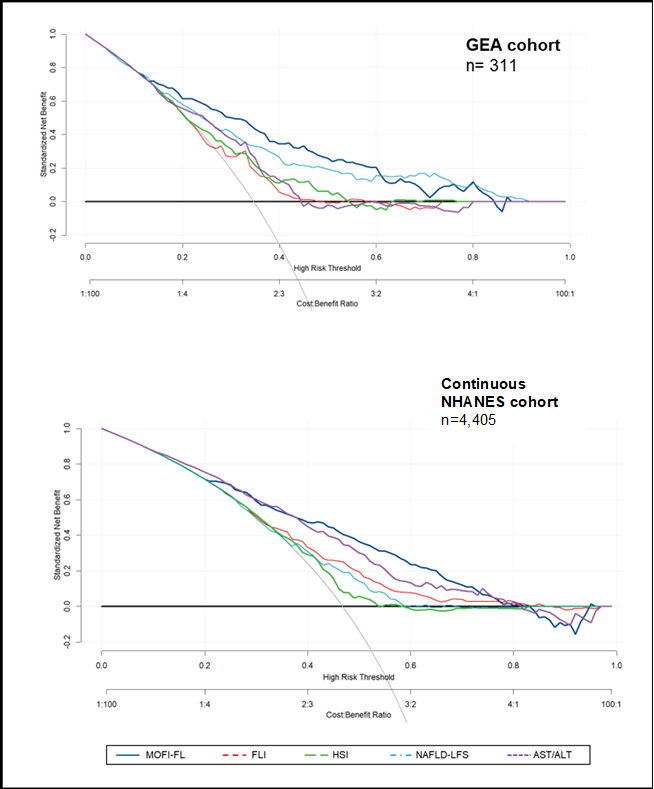


**Supplementary Table 1**: Methodological details of the included cohorts.

| **Cohort name, recruitment years and study center** | **Original objective** | **Study Desing** | **Population and original sample** | **Original inclusion criteria** | **Definitions** | **Ethics approval number (where applicable)** | **Reference** |
| --- | --- | --- | --- | --- | --- | --- | --- |
| Genetics of Atherosclerotic Disease (GEA) study, 2008 to 2012– National Institute of Cardiology Ignacio Chávez, Mexico City | A community-based cohort of adults designed to investigate the genomic basis of premature coronary artery disease and its relationship with traditional and emerging risk factors in the Mexican population | Prospective Cohort | - Mexican-Mestizo (n=2,800) - Convenience sampling | Mexican-Mestizo (with parents and grandparents born in Mexico). Women and men aged 30-75 with a BMI between 18 and 39.9 kg/m^2^ recruited from donors attending the National Institute of Cardiology Ignacio Chavez blood bank or recruited by advertisement posters in social service centers from June 2008 through November 2012. | In the GEA study, it was defined by a liver-to-spleen attenuation ratio (L:S) of < 1.0 measured with computed tomography | The Ethics and Research Committee of Instituto Nacional de Cardiología Ignacio Chavez (No. 09–646). | (1) |
| National Health and Nutrition Survey (continuous NHANES), cycle 2017-2018, National Center for Health Statistics, United States | To address emerging public health issues in the United States | Cross-Sectional | - U.S residents (multiethnic, n= 19,643) - Nationally representative sample of the civilian non-institutionalized US population | The present study included participants of both sex from NHANES 2017–2018 who were 20 years old. Exclusion criteria were as follows: individuals with ineligible study, not performed, or partial elastography examination status; missing controlled attenuation parameter (CAP, which can quantify the steatosis degree) data; individuals with reported status of hepatitis B  or hepatitis C; individuals  with significant alcohol consumption (individuals  who drink an average of one to two or more  standardized drinks per day, respectively) or missing information  about alcohol consumption; taking steatogenic drugs (such as amiodarone, methotrexate,  and tamoxifen) for at least 3 months before  study recruitment; and (6) missing information  regarding any variables required for index calculation. | In the continuous NHANES cohort, when controlled attenuation parameter (CAP) value > 285 dB/m. | The Research Ethics Review Board of NCHS approved the NHANES study | (2) |
| National Health and Nutrition Survey (NHANES) III, 1988-1994 National Center for Health Statistics, United States | Same as continuous NHANES | Cross-Seccional inked to the National Death Index (NDI) | U.S residents (multiethnic, n= 36,054) | The present study included participants of both genders from NHANES III (1988-1994) who were 20 years old. The participants were excluded if were as missing information  regarding any variables required for index calculation. | NA | NHANES III underwent approval by the NCHS Research Ethics Review Board, and all participants gave informed consent. | (3,4) |

Supplementary table 2. STARD checklist

|  | **Section & Topic** | **No** | **Item** | **Reported on page #** |
| --- | --- | --- | --- | --- |
|  |  |  |  |  |
|  | **TITLE OR ABSTRACT** |  |  |  |
|  |  | **1** | Identification as a study of diagnostic accuracy using at least one measure of accuracy  (such as sensitivity, specificity, predictive values, or AUC) | 1 |
|  | **ABSTRACT** |  |  |  |
|  |  | **2** | Structured summary of study design, methods, results, and conclusions  (for specific guidance, see STARD for Abstracts) | 4 |
|  | **INTRODUCTION** |  |  |  |
|  |  | **3** | Scientific and clinical background, including the intended use and clinical role of the index test | 5-6 |
|  |  | **4** | Study objectives and hypotheses | 5-6 |
|  | **METHODS** |  |  |  |
|  | *Study design* | **5** | Whether data collection was planned before the index test and reference standard  were performed (prospective study) or after (retrospective study) | 6-7 |
|  | *Participants* | **6** | Eligibility criteria | Supplementary table 1 |
|  |  | **7** | On what basis potentially eligible participants were identified  (such as symptoms, results from previous tests, inclusion in registry) | Supplementary table 1 |
|  |  | **8** | Where and when potentially eligible participants were identified (setting, location and dates) | Supplementary table 1 |
|  |  | **9** | Whether participants formed a consecutive, random or convenience series | Supplementary table 1 |
|  | *Test methods* | **10a** | Index test, in sufficient detail to allow replication | 8 |
|  |  | **10b** | Reference standard, in sufficient detail to allow replication | 8 |
|  |  | **11** | Rationale for choosing the reference standard (if alternatives exist) | 5-10 |
|  |  | **12a** | Definition of and rationale for test positivity cut-offs or result categories  of the index test, distinguishing pre-specified from exploratory | 5-10 |
|  |  | **12b** | Definition of and rationale for test positivity cut-offs or result categories  of the reference standard, distinguishing pre-specified from exploratory | 5-10 |
|  |  | **13a** | Whether clinical information and reference standard results were available  to the performers/readers of the index test | 5-10 |
|  |  | **13b** | Whether clinical information and index test results were available  to the assessors of the reference standard | 5-10 |
|  | *Analysis* | **14** | Methods for estimating or comparing measures of diagnostic accuracy | 5-10 |
|  |  | **15** | How indeterminate index test or reference standard results were handled | 5-10 |
|  |  | **16** | How missing data on the index test and reference standard were handled | 5-10 |
|  |  | **17** | Any analyses of variability in diagnostic accuracy, distinguishing pre-specified from exploratory | 5-10 |
|  |  | **18** | Intended sample size and how it was determined | 5-10 |
|  | **RESULTS** |  |  |  |
|  | *Participants* | **19** | Flow of participants, using a diagram | Figure 1 |
|  |  | **20** | Baseline demographic and clinical characteristics of participants | 10-15 |
|  |  | **21a** | Distribution of severity of disease in those with the target condition | 10-15 |
|  |  | **21b** | Distribution of alternative diagnoses in those without the target condition | 10-15 |
|  |  | **22** | Time interval and any clinical interventions between index test and reference standard | 10-15 |
|  | *Test results* | **23** | Cross tabulation of the index test results (or their distribution)  by the results of the reference standard | 10-15 |
|  |  | **24** | Estimates of diagnostic accuracy and their precision (such as 95% confidence intervals) | 10-15 |
|  |  | **25** | Any adverse events from performing the index test or the reference standard | 10-15 |
|  | **DISCUSSION** |  |  |  |
|  |  | **26** | Study limitations, including sources of potential bias, statistical uncertainty, and generalisability | 15-18 |
|  |  | **27** | Implications for practice, including the intended use and clinical role of the index test | 15-18 |
|  | **OTHER INFORMATION** |  |  |  |
|  |  | **28** | Registration number and name of registry | Supplementary table 1 |
|  |  | **29** | Where the full study protocol can be accessed | 19 |
|  |  | **30** | Sources of funding and other support; role of funders | 19 |
|  |  |  |  |  |

**Supplementary table 3.**

Description of the measuring techniques for biochemical and anthropometric variables included in GEA study.

| *Characteristics* | *Measurement* | *Measuring technique* |
| --- | --- | --- |
| Anthropometric | Weight (kg) | Weight is measured on a digital scale with the subject standing still in the center of the scale platform facing the recorder, hands at side, and looking straight ahead. The measurement is recorded in kilograms. |
|  | Height (cm) | Height is measured using a stadiometer with the subject standing upright, barefoot, and looking straight ahead. The heels are placed together, arms rest at the sides, and the back is positioned against the stadiometer. The measurement is recorded in centimeters. |
|  | Waist circumference (cm) | With the patient's standing position, waist circumference was measured at the midpoint between the top of the iliac crest and the lower margin of the last palpable rib in the midpoint line. |
|  | BMI (kg/m^2^) | Calculated by dividing the body weight (in kilograms) by the squared body height (in meters). |
|  | Visceral fat area (cm^2^) | Visceral adipose tissue (VAT) was quantified using computed tomography (CT) scans. CT images were acquired using a 64-channel helical multidetector tomograph (Somatom Sensation, Siemens, Malvern, PA, USA). The analysis was performed on a Leonardo workstation (Siemens, Forchheim, Germany) equipped with a specialized fat analysis program. VAT was measured using a threshold attenuation range between −190 and −30 Hounsfield Units (HU). |
|  | Hepatic Steatosis | Multidetector computed tomography is a validated method for identifying the presence of fatty liver. Fatty liver was defined by a liver-to-spleen attenuation ratio <1.0, using a 64-slice scanner (Somatom Cardiac Sensation; Medical Solutions, Forchheim, Germany). |
| Biochemical | Glucose (mg/dL) | Venous blood samples were collected from subjects after 12 hours of fasting and 20 minutes in a sitting position. Glucose levels were determined using an enzymatic colorimetric method. (Roche Diagnostics  GmbH, Mannheim, Germany) |
|  | Insulin (µU/mL) | Venous blood samples were collected from subjects after 12 hours of fasting and 20 minutes in a sitting position. Insulin concentrations were measured using a radioimmunoassay (RIA). |
|  | Uric acid (mg/dL) | Venous blood samples were collected from subjects after 12 hours of fasting and 20 minutes in a sitting position. Uric acid levels were measured using an enzymatic method. (Roche Diagnostics  GmbH, Mannheim, Germany) |
|  | Triglycerides (mg/dL) | Venous blood samples were collected from subjects after 12 hours of fasting and 20 minutes in a sitting position. Triglyceride levels were determined using an enzymatic colorimetric method. (Roche Diagnostics  GmbH, Mannheim, Germany) |
|  | Total cholesterol (mg/dL) | Venous blood samples were collected from subjects after 12 hours of fasting and 20 minutes in a sitting position. Total cholesterol concentrations were measured using an enzymatic method. (Roche Diagnostics  GmbH, Mannheim, Germany) |
|  | HDL-C (mg/dL) | Venous blood samples were collected from subjects after 12 hours of fasting and 20 minutes in a sitting position. High-density lipoprotein cholesterol (HDL-C) was quantified using a direct enzymatic method. (Roche Diagnostics  GmbH, Mannheim, Germany) |
|  | LDL-C (mg/dL) | Venous blood samples were collected from subjects after 12 hours of fasting and 20 minutes in a sitting position. Low-density lipoprotein cholesterol (LDL-C) levels were calculated using the DeLong formula  (LDL-C = Total cholesterol−HDL-C −(Triglycerides/5)) |
|  | AST (U/L) | Venous blood samples were collected from subjects after 12 hours of fasting and 20 minutes in a sitting position. Aspartate aminotransferase (AST) activity was measured using an automated enzymatic assay. (Roche Diagnostics  GmbH, Mannheim, Germany) |
|  | ALT (U/L) | Venous blood samples were collected from subjects after 12 hours of fasting and 20 minutes in a sitting position. Alanine aminotransferase (ALT) levels were measured using an automated enzymatic method. |
|  | GGT (U/L) | Venous blood samples were collected from subjects after 12 hours of fasting and 20 minutes in a sitting position. Gamma-glutamyl transferase (GGT) activity was determined using an automated colorimetric assay. (Roche Diagnostics  GmbH, Mannheim, Germany) |
|  | Adiponectin (µg/mL) | Venous blood samples were collected from subjects after 12 hours of fasting and 20 minutes in a sitting position. Human total adiponectin (ADPN) levels were determined using a Quantikine ELISA kit (R&D Systems, Minneapolis, MN). |
|  | hs-CRP (mg/L) | Venous blood samples were collected from subjects after 12 hours of fasting and 20 minutes in a sitting position. High-sensitivity C-reactive protein (hs-CRP) levels were determined by immunonephelometric. |

**Suplementary Table 4.** R packages used for the statistical analyses

| **R Statistical Package (Version)** | **Rationale of the implementation of the package** | **Citation** |
| --- | --- | --- |
| *mice* (Version 3.14.0) | To generate five imputed datasets, carrying out a maximum of five iterations and combining them according to Rubin's rules. | (5) |
| *glmult* (Version 1.0.8). | To identify the best predictive model for HS in the discovery sample. | (6) |
| *caret* (Version 7.0-1) | To perform a 10-cross fold validation. | (7) |
| *rmda* (Version 1.6) | To perform decision curves to quantify the net clinical benefit of MOFI-FL across different threshold probabilities. | (8) |
| *pROC* (Version 1.18.5) | To perform area under the receiver operating characteristic curve (AUROC). | (9) |
| *dcurves* (Version 0.5.0) | To assess the clinical utility and net benefit of the MOFI-FL index compared to other HS indices and their individual components. | (10) |
| *survival* (Version 3.8-3) | To perform Cox proportional hazards regression models adjusted by age, sex, ethnicity, and number of chronic comorbidities. | (11) |

**Supplementary Table 5.** Selection of predictive variables for HS: top 10 best ranked models from exhaustive search method in the training set

|  | Variables include in the model | AIC |
| --- | --- | --- |
| 1 | ALT_log_ + GGT_log_+ Glucose_log_ + Uric acid + BMI_log_+ Adiponectin_log_ + hs-CRP_log_ + Insulin_log_ | 1,311 |
| 2 | Sex + ALT_log_ + GGT_log_+ Glucose_log_ + WC + Uric acid + Adiponectin_log_ + hs-CRP_log_ + Insulin_log_ | 1,311 |
| 3 | AST_log_ + ALT_log_ + GGT_log_ + Glucose_log_ + Uric acid + BMI_log_+ Adiponectin_log_+ hs-CRP_log_ + Insulin_log_ | 1,312 |
| 4 | Sex + ALT_log_ + GGT_log_+ Glucose_log_ + Uric acid + BMI_log_+ Adiponectin_log_+ hs-CRP_log_ + Insulin_log_ | 1,312 |
| 5 | ALT_log_ + GGT_log_+ Glucose_log_ + Triglycerides_log_ + Uric acid + BMI_log_+ Adiponectin_log_+ hs-CRP_log_ + Insulin_log_ | 1,312 |
| 6 | ALT_log_ + GGT_log_+ Glucose_log_ + WC + Uric acid + BMI_log_+ Adiponectin_log_+ hs-CRP_log_ + Insulin_log_ | 1,312 |
| 7 | ALT_log_ + GGT_log_+ Glucose_log_ + Uric acid + BMI_log_+ Adiponectin_log_+ hs-CRP_log_ + Insulin_log_ + CLDL | 1,312 |
| 8 | Sex + AST_log_+ ALT_log_ + GGT_log_+ Glucose_log_ + WC + Uric acid + Adiponectin_log_+ hs-CRP_log_ + Insulin_log_ | 1,312 |
| 9 | ALT_log_ + GGT_log_+ Glucose_log_ + WC + Uric acid + BMI_log_+ Adiponectin_log_+ hs-CRP_log_ + Insulin_log_ | 1,313 |
| 10 | Sex + ALT_log_ + GGT_log_ + Glucose_log_ + Triglycerides_log_ + WC + Uric acid + Adiponectin_log_+ hs-CRP_log_ + Insulin_log_ | 1,313 |

AST: aspartate aminotransferase, ALT: alanine aminotransferase, GGT: Gamma-glutamyl transferase, BMI: Body mass index, hs-CRP: High-sensitivity C-reactive protein, CLDL: Cholesterol in low-density lipoprotein, AIC: Akaike Information Criterion.

**Supplementary Table 6**. Logistic regression model to identify NAFLD, used for the development of the MOFI-FL.

|  | **Parameter** | **Estimate** | **95% CI** | **S.E.** | **Z-score** | **p-value** |
| --- | --- | --- | --- | --- | --- | --- |
| *AUC= 0.793 95%CI (0.76-0.82)*  *Nagelkerke r² = 0.42*  *Hosmer Lemeshow p-value= 0.99*  *BIC =335* | Intercept | -25.34 | -29.73, -20.96 | 2.20 | -11.47 | <0.0001 |
|  | ALT_In_ (U/L) | 1.56 | 1.25, 1.86 | 0.15 | 10.04 | <0.0001 |
|  | Uric acid (mg/dL) | 0.18 | 0.08, 0.27 | 0.04 | 3.84 | <0.0001 |
|  | Glucose _In_ (mg/dL) | 1.11 | 0.53, 1.68 | 0.29 | 3.82 | <0.0001 |
|  | BMI _In_ (kg/m^2^) | 3.93 | 2.89, 4.96 | 0.52 | 7.44 | <0.0001 |

BMI: body mass index, ALT: alanine aminotransferase, S.E.: standard error

**Supplementary Table 7.** Example for predicting the probability of hepatic steatosis using MOFI-FL

To predict the probability of fatty liver using the MOFI-FL, you need to apply a formula based on clinical and biochemical parameters. This involves inserting individual values into the model equation. Here’s an example of how to apply it:

Patient Background: A male with no history of significant alcohol consumption presents for metabolic evaluation. During the clinical and biochemical assessment, the following values were obtained: ALT (alanine aminotransferase) at 70 U/L, uric acid at 6.5 mg/dL, fasting plasma glucose at 110 mg/dL, and a BMI of 28 kg/m².

We first, obtain the values that are considering for each individual component of the MOFI-FL equation as follows:

- _ln_(ALT) (alanine aminotransferase): 4.248 (which is ln(70))
- Uric acid: 6.5 mg/dL
- _ln_(Glucose): 4.700 (which is ln(110))
- _ln_(BMI): 3.330 (which is ln(28))

Then, we extract the MOFI-FL coefficients as follows:

- Intercept (β0): -25.36568
- _ln_(ALT) coefficient (β1): 1.56099
- Uric acid coefficient (β2): 0.18511
- _ln_(Glucose) coefficient (β3): 1.11809
- _ln_(BMI) coefficient (β4): 3.92416

Step-by-Step Calculation

1. Step 1: Multiply the values by their respective coefficients:

- _ln_(ALT): 4.248×1.56099=6.6284
- Uric acid: 6.5×0.18511=1.2036
- _ln_(Glucose): 4.700×1.11809=5.2504
- _ln_(BMI): 3.330×3.92416=13.0633

1. Step 2: Add the intercept and sum the results from Step 1:

- Sum= −25.36568 + 6.628 + 1.203 + 5.251 + 13.063 = 0.778

1. Step 3: Convert the result into a probability using the logistic function:

$$P=\frac{ⅇ^{sum}}{1+ⅇ^{sum}}*100$$

Substituting the sum:

$$P=\frac{ⅇ^{0.778}}{1+ⅇ^{0.778}}*100= \frac{2.179}{3.179}*100=68.5$$

Final Probability

The individual has a 68.5% probability of having hepatic steatosis based on the MOFI-FL index.

A simplified approach could be implemented using the Shinny App available at: <https://juanreyesbarrera.shinyapps.io/MOFI-FL/>


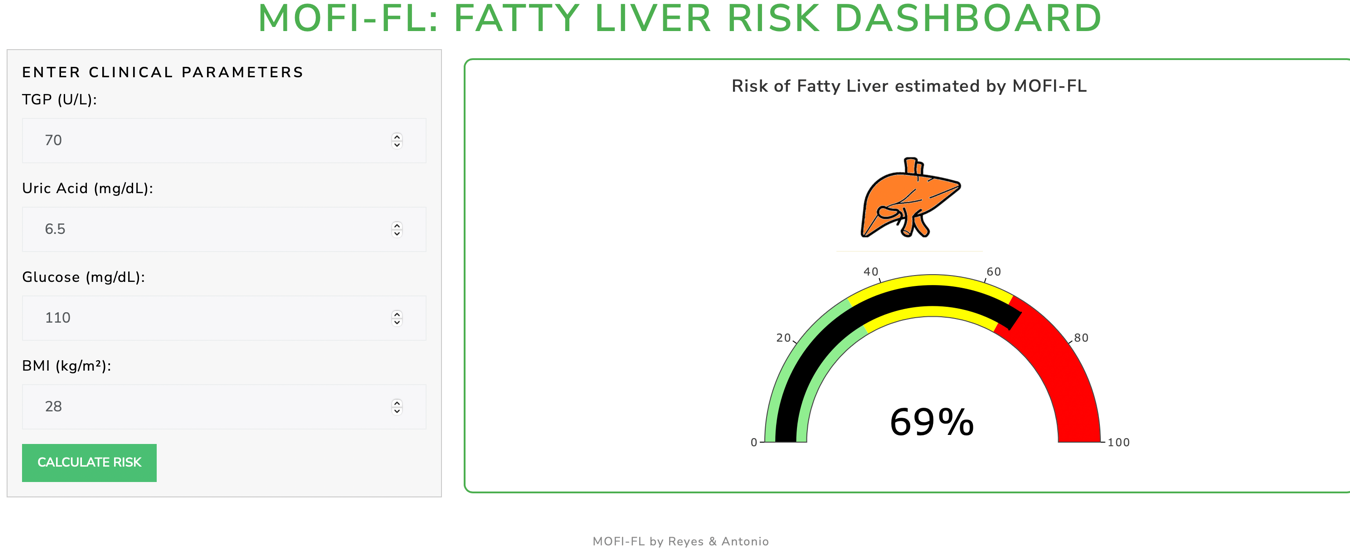


|  |  |  | **Discrimination** | | | | | | | |
| --- | --- | --- | --- | --- | --- | --- | --- | --- | --- | --- |
| *Cohort* | *Model* |  | *Accuracy %* | *Sensitivity%* | *Speciﬁcity%* | *PPV%* | *NPV%* | *LR+* | *LR-* | *AUC* |
| ***GEA***  ***internal validation***  ***cohort*** | **MOFI-FL** |  | **74 (69,79)** | **50 (39,58)** | **88 (82,91)** | **68 (56,78)** | **76 (70,81)** | **3.9 (3.0,5.0)** | **0.59 (0.45,0.77)** | **0.78 (0.72,0.83)** |
|  | Glucose |  | 65 (59, 70) | 8 (4,14) | 95 (92,98) | 47 (25,71) | 66 (61,72) | 1.7 (0.6, 4.5) | 0.96 (0.89,1.02) | 0.65 (0.58,0.71) |
|  | Uric acid |  | 68 (62, 72) | 23 (15,32) | 91 (87,95) | 58 (43,73) | 69 (63,74) | 2.6 (1.4, 4.9) | 0.84 (0.75,0.94) | 0.65 (0.58,0.71) |
|  | ALT |  | 72 (67, 77) | 38 (28,48) | 90 (86,94) | 67 (56,78) | 73 (68,78) | 3.8 (2.4, 6.3) | 0.69 (0.58,0.80) | 0.73 (0.67,0.79) |
|  | BMI |  | 65 (59, 70) | 14 (8,21) | 92 (88,95) | 47 (30,65) | 67 (61,72) | 1.6 (0.8, 3.2) | 0.94 (0.86,1.02) | 0.67 (0.62,0.73) |
| Continuous NHANES  **cohort** | **MOFI-FL** |  | **70** (68,71) | **73** (71) | 68 (66,70) | 57 (55,59) | **81**(79,83) | **2.3 (2.2,2.3)** | **0.40 (0.3,0.40)** | **0.77 (0.76,0.78)** |
|  | Glucose |  | 62 (60, 63) | 41 (38, 43) | 81 (79,82) | 65 (63,68) | 60 (59,62) | 2.1 (1.9,2.4) | 0.73 (0.70,0.76) | 0.66 (0.65,0.68) |
|  | Uric acid |  | 55 (54, 57) | 34 (31,36) | 75 (73,76) | 54 (51,57) | 56 (54,58) | 1.3 (1.2,1.5) | 0.88 (0.84,0.91) | 0.58 (0.57,0.60) |
|  | ALT |  | 59 (58, 61) | 41 (39,43) | 75 (74,77) | 60 (57,62) | 59 (57,61) | 1.7 (1.5,1.8) | 0.77 (0.74,0.80) | 0.62 (0.61,0.64) |
|  | BMI |  | 67 (65, 68) | 60 (58,62) | 73 (71,75) | 66 (64,68) | 67 (65,69) | 2.2 (2.1,2.4) | 0.54 (0.51,0.57) | 0.74 (0.73,0.76) |

**Supplementary Table 8**. Evaluation of the performance of MOFI-FL and its components in identifying HS.

Values in parentheses represent the 95% confidence interval (CI). MOFI-FL: model of identification of fatty liver, ALT: alanine aminotransferase, BMI: body mass index, PPV: positive predictive value, NPV: negative predictive value, LR+: positive likelihood ratio, LR-: negative likelihood ratio, AUC: area under the roc curve.

|  | **Overall,**  n= 12,684^1^ | **Low risk**  **<25**,  n = 9,975^1^ | **Medium risk**  **26-50**,  n = 1,716^1^ | **High risk**  **51-75**,  n = 739^1^ | **Very high risk**  **>75**,  n = 254^1^ |
| --- | --- | --- | --- | --- | --- |
| *Clinical characteristics* |  |  |  |  |  |
| Age (years) | 49 ± 20 | 49 ± 20 | 48 ± 17 | 46 ± 15 | 41 ± 14 |
| Sex, female n (%) | 6658 (53) | 5611 (84) | 666 (10) | 279 (4) | 102 (2) |
| BMI (Kg/m^2^) | 27 ± 6 | 26 ± 4 | 32 ± 5 | 34 ± 7 | 37 ± 9 |
| Glucose(mg/dL) | 100 ± 38 | 95 ± 26 | 115 ± 54 | 124 ± 63 | 141 ± 83 |
| ALT (U/L) | 18 ± 16 | 13 ± 6 | 25 ± 13 | 39 ± 24 | 75 ± 59 |
| Uric Acid(mg/dL) | 5.4 ± 1.5 | 5.1 ± 1.4 | 6.2 ± 1.4 | 6.6 ± 1.6 | 6.8 ± 1.8 |
| *Mortality causes* |  |  |  |  |  |
| All causes of mortality, n (%) | 5619 (44) | 5,563 (56) | 960 (56) | 402 (54) | 146 (57) |
| Cardiovascular, n (%) | 1764 (14) | 1,404 (14) | 219 (13) | 116 (16) | 25 (9.8) |
| Cerebrovascular diseases, n (%) | 382 (3) | 309 (3.1) | 50 (2.9) | 19 (2.6) | 4 (1.6) |
| Malignant neoplasms, n (%) | 1220(9) | 975 (9.8) | 178 (10) | 51 (6.9) | 16 (6.3) |
| Diabetes mellitus, n (%) | 190 (1.5) | 103 (1.0) | 46 (2.7) | 29 (3.9) | 12 (4.7) |
| Respiratory diseases, n (%) | 235 (1.8) | 199 (2.0) | 22 (1.3) | 12 (1.6) | 2 (0.8) |
| Nephrotic diseases, n (%) | 89 (0.7) | 70 (0.7) | 11 (0.6) | 6 (0.8) | 2 (0.8) |
| Months of follow-Up | 262 ±110 | 261 ± 113 | 269 ± 101 | 266 ± 100 | 270 ± 101 |
| *Ethnicity* |  |  |  |  |  |
| Caucasic, n (%) | 5491 (43) | 4596 (84) | 594 (11) | 231 (4) | 70 (1) |
| Afro-American, n (%) | 3344 (27) | 2593 (77) | 500 (15) | 199 (6) | 52 (2) |
| Mexican American, n (%) | 3381(27) | 2431 (72) | 555 (16) | 271 (8) | 124 (4) |
| Other ethnic, n (%) | 448 (3) | 335 (75) | 67 (15) | 38 (8) | 8 (2) |
| *Medical history* |  |  |  |  |  |
| Hypertension, n (%) | 3533 (28) | 2477 (25) | 631 (37) | 316 (43) | 109 (43) |
| Arthritis, n (%) | 2822 (22) | 2204 (22) | 389 (23) | 180 (24) | 49 (19) |
| Asthma, n (%) | 872 (7) | 666 (7) | 132 (8) | 54 (7) | 20 (8) |
| Diabetes, n (%) | 1057 (8) | 628 (6) | 241 (14) | 140 (19) | 48 (19) |
| Bronchitis, n (%) | 749 (6) | 589 (6) | 95 (6) | 48 (7) | 17 (7) |
| Malignancy, n (%) | 505 (4) | 437 (4) | 47 (3) | 14 (2) | 7 (3) |
| Heart attack, n (%) | 612 (5) | 473 (5) | 78 (5) | 51 (7) | 10 (4) |
| Stroke, n (%) | 377 (3) | 307 (3) | 50 (3) | 16 (2) | 4 (2) |
| Emphysema, n (%) | 268 (2) | 235 (2) | 18 (1) | 13 (2) | 2 (1) |
| ≥1 Comorbidity, n (%) | 3377 (27) | 4757 (48) | 979 (57) | 439 (59) | 150 (59) |
| ^1^Mean ± SD; n (%) |  | | | | |

**Supplementary Table 9**. NHANES III population characteristics by categories of MOFI-FL risk

# Supplementary Table 10. Prediction of cause-specific mortality risk using MOFI-FL, FLI, HSI,NAFLD and AST/ALT (n=8,562)

| **Outcome** | **Model** | **MOFI-FL**  **HR (95% CI)** | **FLI**  **HR (95% CI)** | **HSI**  **HR (95% CI)** | **NAFLD-LFS**  **HR (95% CI)** | **AST/ALT**  **HR (95% CI)** |
| --- | --- | --- | --- | --- | --- | --- |
| All causes | 1 | 1.086 (1.047, 1.127) | 1.199 (1.163, 1.237) | 0.987 (0.949, 1.026) | 1.100 (1.078, 1.123) | 1.052 (1.030, 1.073) |
|  | 2 | 1.045 (1.007, 1.085) | 1.163 (1.127, 1.201) | 0.946 (0.910, 0.984) | 1.074 (1.049, 1.101) | 1.059 (1.038, 1.081) |
| Cardiovascular | 1 | 1.137 (1.064, 1.215) | 1.181 (1.114, 1.252) | 1.044 (0.971, 1.122) | 1.111 (1.075, 1.149) | 1.055 (1.019, 1.092) |
|  | 2 | 1.057 (0.988, 1.132) | 1.108 (1.043, 1.178) | 0.971 (0.904, 1.043) | 1.069 (1.023, 1.117) | 1.070 (1.034, 1.107) |
| Diabetes | 1 | 1.927 (1.661, 2.235) | 1.765 (1.544, 2.018) | 1.687 (1.395, 2.039) | 1.220 (1.181, 1.261) | 0.579 (0.412, 0.815) |
|  | 2 | 1.682 (1.441, 1.964) | 1.588 (1.376, 1.832) | 1.427 (1.177, 1.731) | 1.187 (1.145, 1.231) | 0.665 (0.476, 0.930) |
| Nephrotic diseases | 1 | 1.278 (0.993, 1.646) | 0.986 (0.726, 1.339) | 0.956 (0.703, 1.302) | 1.186 (1.098, 1.281) | 0.982 (0.753, 1.280) |
|  | 2 | 1.193 (0.920, 1.546) | 0.906 (0.658, 1.249) | 0.888 (0.654, 1.206) | 1.164 (1.066, 1.270) | 1.010 (0.793, 1.287) |
| Cerebrovascular  diseases | 1 | 1.133 (0.984, 1.304) | 1.247 (1.109, 1.402) | 1.073 (0.926, 1.242) | 1.051 (0.939, 1.178) | 1.027 (0.941, 1.120) |
|  | 2 | 1.089 (0.942, 1.258) | 1.214 (1.076, 1.370) | 1.030 (0.888, 1.194) | 1.006 (0.877, 1.155) | 1.037 (0.952, 1.128) |
| Malignant neoplasms | 1 | 0.936 (0.861, 1.017) | 1.080 (1.006, 1.159) | 0.904 (0.832, 0.982) | 1.016 (0.944, 1.094) | 1.059 (1.014, 1.105) |
|  | 2 | 0.924 (0.849, 1.006) | 1.071 (0.997, 1.150) | 0.891 (0.820, 0.969) | 1.002 (0.925, 1.084) | 1.060 (1.015, 1.106) |
| Chronic lower  respiratory diseases | 1 | 0.830 (0.666, 1.034) | 1.185 (1.016, 1.382) | 0.847 (0.693, 1.034) | 0.837 (0.660, 1.062) | 1.040 (0.932, 1.160) |
|  | 2 | 0.777 (0.621, 0.972) | 1.122 (0.957, 1.316) | 0.801 (0.658, 0.975) | 0.743 (0.587, 1.042) | 1.050 (0.944, 1.169) |

Model 1: adjusted by age and sex.

Model 2: Model 1 plus ethnicity and number of comorbidities

| Index | C-index  Univariate | C-index  Model 1 | Δ C-index  Model 1 – Univariate | C-index  Model 2 | Δ C-index  Model 2 – Univariate |
| --- | --- | --- | --- | --- | --- |
| MOFI-FL | 0.505 (0.500,0.509) | 0.841 (0.835,0.846) | 0.336 | 0.845 (0.839,0.850) | 0.340 |
| FLI | 0.429 (0.420, 0.431) | 0.844 (0.838,0.849) | 0.415 | 0.848 (0.842,0.853) | 0.419 |
| HSI | 0.452 (0.447. 0.461) | 0.840 (0.834,0.845) | 0.388 | 0.845 (0.839,0.850) | 0.393 |
| NAFLD-LFS | 0.470 (0.462,0.481) | 0.841(0.835,0.846) | 0.371 | 0.845 (0.839,0.850) | 0.375 |
| AST/ALT | 0.378 (0.364,0.386) | 0.841 (0.835,0.846) | 0.463 | 0.846 (0.840,0.851) | 0.468 |

**Supplementary Table 11**. C-index of MOFI-FL and other indices

Model 1: Adjusted by age and sex.

Model 2: Model 1 plus ethnicity and number of comorbidities

**Reference**

1. Villarreal-Molina T, Posadas-Romero C, Romero-Hidalgo S, Antúnez-Argüelles E, Bautista-Grande A, Vargas-Alarcón G, et al. The ABCA1 gene R230C variant is associated with decreased risk of premature coronary artery disease: the genetics of atherosclerotic disease (GEA) study. PLoS One. 2012;7(11):e49285.

2. NHANES 2017-2018 Overview [Internet]. [cited 2024 Dec 15]. Available from: https://wwwn.cdc.gov/nchs/nhanes/continuousnhanes/overview.aspx?BeginYear=2017

3. NCHS Data Linkage - Mortality Data [Internet]. 2024 [cited 2024 Dec 15]. Available from: https://www.cdc.gov/nchs/data-linkage/mortality.htm

4. NHANES III (1988-1994) [Internet]. [cited 2024 Nov 30]. Available from: https://wwwn.cdc.gov/nchs/nhanes/nhanes3/default.aspx

5. Buuren S van, Groothuis-Oudshoorn K, Vink G, Schouten R, Robitzsch A, Rockenschaub P, et al. mice: Multivariate Imputation by Chained Equations [Internet]. 2024 [cited 2025 Jan 5]. Available from: https://cran.r-project.org/web/packages/mice/index.html

6. Calcagno V. glmulti: Model Selection and Multimodel Inference Made Easy [Internet]. 2020 [cited 2024 Dec 13]. Available from: https://cran.r-project.org/web/packages/glmulti/index.html

7. Kuhn [aut M, cre, Wing J, Weston S, Williams A, Keefer C, et al. caret: Classification and Regression Training [Internet]. 2024 [cited 2024 Dec 13]. Available from: https://cran.r-project.org/web/packages/caret/index.html

8. Brown M. rmda: Risk Model Decision Analysis [Internet]. 2018 [cited 2024 Dec 13]. Available from: https://cran.r-project.org/web/packages/rmda/index.html

9. Robin X, Turck N, Hainard A, Tiberti N, Lisacek F, Sanchez JC, et al. pROC: Display and Analyze ROC Curves [Internet]. 2023 [cited 2024 Dec 13]. Available from: https://cran.r-project.org/web/packages/pROC/index.html

10. Decision Curve Analysis for Model Evaluation [Internet]. [cited 2025 Jan 5]. Available from: https://www.danieldsjoberg.com/dcurves/

11. Therneau TM, until 2009) TL (original S >R port and R maintainer, Elizabeth A, Cynthia C. survival: Survival Analysis [Internet]. 2024 [cited 2024 Dec 13]. Available from: https://cran.r-project.org/web/packages/survival/index.html
